# Effects of Dispersants and Biosurfactants on Crude-Oil Biodegradation and Bacterial Community Succession

**DOI:** 10.3390/microorganisms9061200

**Published:** 2021-06-01

**Authors:** Gareth E. Thomas, Jan L. Brant, Pablo Campo, Dave R. Clark, Frederic Coulon, Benjamin H. Gregson, Terry J. McGenity, Boyd A. McKew

**Affiliations:** 1 School of Life Sciences, University of Essex, Wivenhoe Park, Essex CO4 3SQ, UK; david.clark@essex.ac.uk (D.R.C.); bgregs@essex.ac.uk (B.H.G.); tjmcgen@essex.ac.uk (T.J.M.); boyd.mckew@essex.ac.uk (B.A.M.); 2 Centre for Environment, Fisheries and Aquaculture Science, Pakefield Road, Lowestoft, Suffolk NR33 0HT, UK; jan.brant@cefas.co.uk; 3 School of Water, Energy and Environment, Cranfield University, Cranfield MK43 0AL, UK; p.campo-moreno@cranfield.ac.uk (P.C.); f.coulon@cranfield.ac.uk (F.C.); 4 Institute for Analytics and Data Science, University of Essex, Wivenhoe Park, Essex CO4 3SQ, UK

**Keywords:** dispersants, biosurfactants, bacteria, OHCB, *Oleispira*, *Pseudomonas*, hydrocarbons, oil

## Abstract

This study evaluated the effects of three commercial dispersants (Finasol OSR 52, Slickgone NS, Superdispersant 25) and three biosurfactants (rhamnolipid, trehalolipid, sophorolipid) in crude-oil seawater microcosms. We analysed the crucial early bacterial response (1 and 3 days). In contrast, most analyses miss this key period and instead focus on later time points after oil and dispersant addition. By focusing on the early stage, we show that dispersants and biosurfactants, which reduce the interfacial surface tension of oil and water, significantly increase the abundance of hydrocarbon-degrading bacteria, and the rate of hydrocarbon biodegradation, within 24 h. A succession of obligate hydrocarbonoclastic bacteria (OHCB), driven by metabolite niche partitioning, is demonstrated. Importantly, this succession has revealed how the OHCB *Oleispira*, hitherto considered to be a psychrophile, can dominate in the early stages of oil-spill response (1 and 3 days), outcompeting all other OHCB, at the relatively high temperature of 16 °C. Additionally, we demonstrate how some dispersants or biosurfactants can select for specific bacterial genera, especially the biosurfactant rhamnolipid, which appears to provide an advantageous compatibility with *Pseudomonas*, a genus in which some species synthesize rhamnolipid in the presence of hydrocarbons.

## 1. Introduction

Oil spills have been one of the primary inputs of pollution into the marine environment since the turn of the 20th century, when large oil tankers became prominent [1]. The extraction, processing, and transportation of oil continues to increase, and new oil fields are regularly being discovered [2]. Despite the COVID-19 pandemic, recent forecasts predict that global production and consumption of oil will return to pre-COVID-19 levels of ~100 million barrels per day by mid-2021 [3]. Thus, oil pollution remains a significant threat to marine ecosystems, tourism, and fisheries.

A key remediation tool in response to oil pollution is the application of dispersants [4]. The active surfactants within dispersants transform oil into small, stable droplets [5,6]. These oil droplets are transported from the surface into the water column, creating an oil-in-water emulsion, increasing oil surface area [6], and thus increasing bioavailability to hydrocarbon-degrading microbes [7]. The Deepwater Horizon oil spill in 2010 stimulated a new wave of research into dispersant use, when approximately 10 million litres of Corexit 9500A/9527 were applied during the incident (35% subsea at the wellhead and 65% on the surface) [8]. The objective during the Deepwater Horizon oil spill was to limit impact to surrounding coastlines, and evidence supports the general success of this strategy, with a delay in oil reaching the surface [9,10]. However, the use of dispersants as a form of oil-spill remediation remains a source of contention. While several studies suggest that dispersants enhance the growth of hydrocarbon-degrading bacteria (HCB) [11,12,13,14] and increase biodegradation [15,16], other studies show that dispersants do not enhance biodegradation [17,18] or may even inhibit the growth of HCB [19,20]. Ultimately, the environmental impact and fate of an oil spill are among the main considerations of the Spill Impact Mitigation Assessment (SIMA, [21]) process during decision making for incident response. Balancing the trade-offs of different scenarios must be considered, for example, between the predicted effects of leaving oil on the water surface, versus dispersing oil into the water column and potentially to the seabed, which is often considered preferential to allowing oil to transfer to coastlines, particularly when these include environmentally sensitive areas.

Dispersants are generally comprised of a mixture of surface-active surfactants (non-ionic (e.g., Tween 80) and anionic (e.g., dioctyl sodium sulfosuccinate), constituting 1–50%) and solvents (e.g., kerosene or glycol ether, constituting >50%) [22], though exact compositions are proprietary information. Many dispersants are used globally, and countries have different products stockpiled in case of an oil spill. Dispersants are widely tested for effectiveness and toxicity, for example, the United Kingdom has approval schemes that authorize the stockpiling and application of dispersants such as Finasol OSR 52, Slickgone NS, and Superdispersant 25 [23].

Additionally, there are many biosurfactants, each with a variety of potential applications [24,25,26], though the cost of producing biosurfactants restricts their application to very specific clean-up operations. However, since biosurfactants are one of the most in-demand biotechnological compounds, promising strategies for lower-cost production are emerging [27]. Oil-spill remediation potential of biosurfactants, as well as their effects on bacterial community composition, remains relatively understudied, and so also merits investigation alongside commonly used commercial dispersants. Biosurfactants are synthesized naturally by many microorganisms and consist of a polar (hydrophilic) moiety and non-polar (hydrophobic) group. Rhamnolipids have either 1 or 2 sugar(s) glycosylated to a β-hydroxy (3-hydroxy) fatty acid [28]. Sophorolipids generally consist of di-saccharide (2-O-β-o-glucopyranosyl-β-D-glucopyranose) derivatives, each linked to a hydroxy fatty acid [29]. Trehalolipids structures are variable but generally consist of a non-ionic acylated derived trehalose sugar glycosylated to a mono-, di-, or tri-fatty acid [30]. Most hydrocarbon-degrading microorganisms are known to produce biosurfactants that assist in the emulsification of hydrocarbons [31,32].

It remains unclear how the different compositions of commercial dispersants and biosurfactants may affect bacterial community composition and abundance, and thus biodegradation rates. Therefore, we tested the effects and efficacy of three of the most widely stockpiled commercial dispersants (Finasol OSR 52 (TOTAL), Slickgone NS (Dasic International Ltd.), and Superdispersant 25 (Oil Technics)) in seawater microcosms. Whilst exact compositions are proprietary information, all three tested dispersants contain between 0.2% and 10% dioctyl sodium sulfosuccinate, varying proportions of non-ionic surfactants, and solvents (e.g., kerosene and 2-butoxyethanol) from <10% to >50% [33,34,35]. Additionally, we tested three biosurfactants, selected based on their range of structures and potential for commercial application: rhamnolipid isolated from *Pseudomonas aeruginosa* (15% active ingredient in 85% distilled water; JBR-215, Jeneil Biosurfactant Company), sophorolipid from the yeast *Candida* spp. (15% active ingredient in 85% distilled water; Sigma), and trehalolipid from *Rhodococcus* sp. strain PML026 (100% active ingredient; [36]). Seawater was sampled from a fully saline, coastal region, at the mouth of the Thames Estuary (see Supplementary Figure S1 and full site description in Materials and Methods). The sampling site is therefore representative of similar coastal regions that are both typically rich in nutrients and often subject to oil contamination and occasional large spills (particularly in the UK and Europe, [37]). Dispersants have been applied in estuaries in the past [38,39], and there is continued research into their sustained use in coastal environments [40]. However, with strict guidelines regarding dispersant toxicity, which could potentially damage sensitive coastal environments, they are not always adopted. Though, in some cases, dispersants could provide a net environmental benefit in coastal areas by diluting oil concentrations impacting the coast, with no long-term toxicity effects to coastal species associated with dispersant application [41]. It has been demonstrated that certain species of hydrocarbon-degrading bacteria will produce enough biosurfactant to reduce interfacial surface tension between oil and water between 24 and 48 h [42,43]. We hypothesized significant positive effects, by those dispersants and biosurfactants that can efficiently decrease the interfacial surface tension between oil and water, on both bacterial growth and hydrocarbon biodegradation. This effect would be seen in the early stages (by day 1) before microbes can synthesize their own surfactants and when macronutrients are not limiting. We additionally aimed to uncover whether different dispersant or biosurfactant formulations may select for different hydrocarbon-degrading communities.

## 2. Materials and Methods

### 2.1. Sampling Site

Seawater was sampled from the Thames Estuary (Supplementary Figure S1), Earls Hope saltmarsh, Stanford le Hope, Essex, UK (51°30′ N, 0°27′ E). The Thames is one of the UK’s largest rivers and has over 50 tributaries that drain a diverse catchment, including agricultural and industrial land, and the most highly urbanized area of the UK. The sampling site is a fully saline, coastal region, at the mouth of the hypernutrified Thames Estuary, which is subject to heavy shipping traffic from the DP World London Gateway (deep-sea container port, opened November 2013) and oil/gas storage and transport facilities. Previously, the sampling site was near two oil refineries (closed in 1999 and 2012). Due to heavy shipping traffic, and the transport and storage of oil, the sampling site is likely exposed to constant inputs of petroleum hydrocarbons. Samples were taken in November 2017 at high tide from the sea surface (13.8 °C, 35.1 PSU, pH 8.39).

### 2.2. Microcosm Design and Sampling

Microcosms were created based on ten treatments (Supplementary Materials Table S1) using sterile 40 mL glass vials with PTFE-lined silicon septa. Treatments were destructively sampled in triplicate over a series of time points (1, 3, 7, 14, 21 days), ensuring samples were temporally independent. Each 40 mL microcosm (excluding seawater only) contained 20 mL of seawater which was supplemented with nutrients (final concentrations of 300 µM NH_4_Cl and 20 µM K_2_HPO_4_); this allowed us to control for nutrient limitation and evaluate microbial response to oil/dispersants in the early phase. The sampling site is subjected to large annual inputs of dissolved inorganic nitrogen (DIN) and dissolved inorganic phosphorous (DIP) from the river Thames [44] and the nutrient loadings are representative of background nitrogen and phosphorous concentrations recorded at the sampling site at other times [45]. The oil (0.1% *v*/*v* final concentration) was a Norwegian Geochemical Standard, North Sea Oil (NSO-1; Norwegian Petroleum Directorate, Stavanger, Norway), that had previously been weathered (distilled at 69 °C, which is the boiling point of hexane and thus removes the most volatile components of crude oil). Oil was added to microcosms by reverse-pipetting and time-zero samples were analysed to ensure consistent oil loadings, which consistently added 45.50 and 4.38 µg mL^−1^ ± 7% of resolvable alkanes and PAHs, respectively. The dispersants were Slickgone NS, Superdispersant 25, and Finasol OSR 52 added to an industry standard (0.005% *v*/*v*), creating a ratio of 20:1 oil to dispersant [46]. The biosurfactants were rhamnolipid, sophorolipid, and trehalolipid, and were also added at a final concentration of 0.005% *v*/*v* to create the same ratio, based on the percentage of active ingredient. Killed controls (seawater and oil microcosms with 1.1049 mM of added HgCl_2_) were also sampled at each time point to monitor abiotic hydrocarbon loss. All microcosms were incubated on a rotary shaker at 100 RPM at 16 °C, replicating summer environmental conditions in the Thames Estuary.

### 2.3. Surface Tension

Surface tension of seawater, and any changes due to the addition of dispersants or biosurfactants, was measured on a KRÜSS Force Tensiometer K6 using the Du Noüy ring method [47]. Briefly, this involved lowering a platinum ring to just below the water’s surface, applying tension until the water’s surface breaks, at which point a measurement of the force required was recorded. To avoid contamination, surface tension was measured in independent seawater microcosms (20 mL), with dispersant or biosurfactants added at the same concentration used in the incubation microcosms.

### 2.4. Hydrocarbon Degradation (GC–MS)

Hydrocarbons were extracted from the seawater microcosms (18 mL, after 2 mL was removed for DNA analysis) by vigorously shaking in 6 mL hexane:dichloromethane (1:1) followed by 30 min in an ultrasonic water bath. Deuterated alkane (nonadecane C_19_d_40_ and triacontane C_30_d_62_ at 10 µg mL^−1^) and PAH (naphthalene-C_10_d_8_ and anthrancene-C_14_d_10_ at 10 µg mL^−1^) internal standards were added to each sample and quantification was performed on an Agilent (Cheadle, UK) 7890A Gas Chromatography system coupled with a Turbomass Gold Mass Spectrometer with a Triple-Axis detector, operating at 70 eV in the positive ion mode, using conditions as previously described by Coulon et al. [45]. External multilevel calibrations were carried out using alkanes (Standard Solution (C_8_–C_40_); Sigma, Welwyn Garden City, UK), methylated-PAHs (1-methylnapthalene, 2-methylanthracene, and 9,10-dimethylanthracene; Sigma), and PAH (QTM PAH Mix; Sigma) standards, the concentrations of which ranged from 1.125 to 18 µg mL^−1^. For quality control, a 2.0 ng µL^−1^ diesel standard solution (ASTM C_12_-C_60_ quantitative, Supelco, Welwyn Garden City, UK) and a 1.0 ng µL^−1^ PAH Mix Standard solution (Supelco) were analysed every 15 samples. Variation in the reproducibility of extraction and quantification of water samples was determined by successive extractions and injections (*n =* 6) of the same sample and estimated to be ± 8%. Extraction efficiency was measured at 89% (10% of this was to account for the 2 mL (of the 20 mL seawater microcosms) removed for DNA extraction after homogenization). All alkanes between C_10_ and C_36_ including pristane and phytane and the following PAHs were quantified (naphthalene; all isomers of methyl-, dimethyl- and trimethyl-naphthalenes; acenaphthylene; acenaphthene; fluorene; phenanthrene; all isomers of methyl- and dimethyl-phenanthrenes/anthracenes; fluoranthene; pyrene; all isomers of methyl- and dimethyl-pyrene; chrysene; all isomers of methyl- and dimethyl-chrysene). Only those hydrocarbons detected are shown in Figure S3.

### 2.5. qPCR Analysis of Bacterial 16S rRNA Genes

At each time point, samples were vigorously shaken to allow dispersion of microcosm contents throughout, prior to 2 mL being sampled for DNA extraction. The 2 mL sample was centrifuged at 21,100× *g* for 20 min, leaving a cell pellet in the collection tube. The 2 mL supernatant was removed and stored for nutrient analysis (see Section 2.6). DNA was extracted from cell pellets using a DNeasy PowerSoil Kit (Qiagen, Manchester, UK), according to the manufacturer’s instructions. DNA extracts were frozen at −20 °C for downstream analysis, including qPCR (to monitor total bacterial biomass) and amplicon library sequencing (to evaluate bacterial community composition). The primers used for quantification of bacterial 16S rRNA gene were 341f—CCTACGGGNGGCWGCAG and 785r—GACTACHVGGGTATCTAATCC [48]. All qPCR reactions were performed using a CFX384™ Real-Time PCR Detection System (BioRad, Watford, UK) with a PCR using reagents, cycle conditions, and standards as previously described [49]. Inspection of standard curves showed that all assays produced satisfactory efficiency (91%) and *R*^2^ values (>0.99).

### 2.6. Nutrient Concentration

Nutrient analysis was conducted on all samples to determine concentrations of ammonium (NH_4_^+^) and phosphate (PO_4_^3−^) using a SEAL Analytical AA3 HR AutoAnalyzer tandem JASCO FP-2020 Plus fluorescence detector.

### 2.7. Amplicon Sequencing and Bioinformatics

Amplicon libraries were prepared, as per Illumina instructions, by a 25-cycle PCR. PCR primers were the same as those used for qPCR but flanked with Illumina Nextera overhang sequences. A unique combination of Nextera XT Indices (Illumina, Cambridge, UK) were added to PCR products from each sample, via an 8-cycle PCR. PCR products were quantified using a Quant-iT™ PicoGreen™ dsDNA Assay Kit (ThermoFisher Scientific, Dartford, UK) and pooled in equimolar concentrations. Quantification of the amplicon libraries was determined via NEBNext^®^ Library Quant Kit for Illumina (New England BioLabs Inc., Hitchin, UK), prior to sequencing on the Illumina MiSeq^®^ platform (Illumina, Cambridge, UK), using a MiSeq^®^ 600 cycle v3 reagent kit and 20% PhiX sequencing control standard. Raw sequence data have been submitted to the European Nucleotide Archive database under accession number PRJEB37243. Sequence output from the Illumina MiSeq platform were analysed within R [50] using an ASV (amplicon sequence variants) bioinformatics pipeline within the DADA2 package [51]. The DADA2 pipeline included primer trimming, quality filtering, error correction and sample inference (using the DADA2 algorithm at default parameters and pooling of samples), and removal of chimeras. Finally, taxonomy assignments for ASVs were obtained using the RDP (Ribosomal Database Project) classifier [52]. 

### 2.8. Phylogenetic Analysis

The Neighbour-Joining protocol [53] was used to infer the evolutionary history of partial 16S rRNA gene sequences bacterial ASVs, aligned with known hydrocarbon-degrading bacteria and closest neighbouring accessions using MUSCLE [54]. Bootstrapping analysis (1000 iterations) was conducted to determine the percentage of time associated taxa clustered together in replicate trees [55]; only bootstrap values >70% are shown. Evolutionary distances, units in the number of base substitutions per site, were calculated with the use of Maximum Composite Likelihood protocol (using the Tamura-Nei model [56]). Phylogenetic analyses were conducted in MEGA7.

### 2.9. Statistical Analysis

Data were first tested for normality (Shapiro–Wilks test); those data which were normally distributed were tested for significance with ANOVAs or appropriate linear models. Non-normally distributed data were analysed using appropriate GLMs (Generalized Linear Models) as follows. The relative abundance of ASVs or genera in relation to oil exposure, dispersant/biosurfactant, and time were modelled using multivariate negative binomial GLMs [57]. Here, the number of sequences in each library was accounted for using an offset term, as described previously [58]. The abundance of bacterial 16S rRNA gene copies was also modelled using negative binomial GLMs [59]. The significance of model terms was assessed via likelihood ratio tests. The Ecological Index of Hydrocarbon Exposure (EIHE) [60] was calculated using the script available at the ecolFudge GitHub page (https://github.com/Dave-Clark/ecolFudge (accessed on 18 May 2020)) [61], and EIHE values modelled using poisson GLMs. Co-occurrence analysis was conducted by network analysis using the SPIEC-EASI algorithm [62]. To make this analysis computationally tractable and biologically interpretable, the total bacterial ASV table was filtered to ASVs confidently (RDP classifier confidence >0.8) assigned to putative hydrocarbon-degrading genera specified in the EIHE [60]. ASVs assigned to several other genera (*Colwellia*, *Arcobacter*, *Glaciecola*, *Marinomonas*, *Zhongshania*, *Neptuniibacter*, and *Alkalimarinus*) that are not included in this index were also included, based on strong evidence for a direct or indirect role in hydrocarbon degradation. For this analysis, unnormalised data were used, as the SPIEC-EASI algorithm applies its own internal log ratio transformation to normalize ASV counts. Network inference was conducted using the neighbourhood selection framework (50 permutations). The network was clustered, using a fast-greedy clustering algorithm, to reveal any modules (smaller groups of ASVs with dense connections within the group, and few between groups). Additionally, the extent to which ASVs co-occurred with other ASVs in the same genus was evaluated by quantifying taxonomic assortativity. An assortativity coefficient quantifies whether nodes in a network tend to connect with other nodes that share properties (such as taxonomy), and ranges from −1 (disassortativity—ASVs never associate with others in the same genus) to 1 (assortativity—ASVs only ever associate with others in the same genus).

All statistical analyses were carried out in R3.6.1 [50] using a variety of packages available through the references [59,63,64,65,66,67,68,69,70,71,72,73]. All plots were constructed using the “ggplot2” [74] and “patchwork” [75] R packages.

## 3. Results

### 3.1. Effects of Dispersants and Biosurfactants on the Surface Tension of Seawater

The addition of dispersants and biosurfactants significantly reduced the surface tension of the seawater (initial value, 69.66 ± 0.24 mN m^−1^; Supplementary Figure S2). There was a reduction in the surface tension of seawater to 60.33 ± 1.25 mN m^−1^ when sophorolipid was added (Supplementary Figure S2), but a much greater effect (coef. = 35.66, *t* = 82.18, *p* < 0.001) was observed with all other dispersants and biosurfactants which reduced the surface tension to between 28 and 34 mN m^−1^.

### 3.2. Effects of Dispersants and Biosurfactants on Hydrocarbon Concentrations

**Figure 1:**
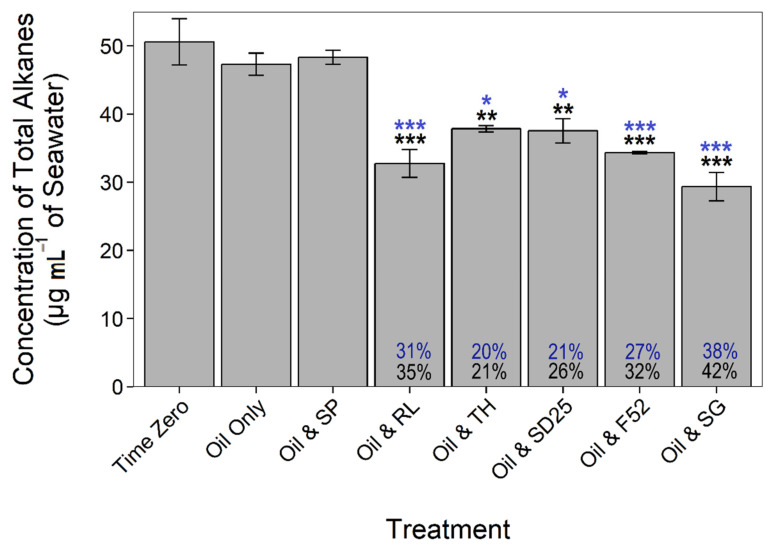
Total alkane concentrations (mean ± SE, *n =* 2) at day 1. Blue (upper) percentages (and asterisks) represent degradation in comparison to the oil-only control. Black (lower) percentages (and asterisks) represent degradation in comparison to starting concentration (“Time Zero”). Dispersant and biosurfactant codes are as follows: SP = sophorolipid, RL = rhamnolipid, TH = trehalolipid, SD25 = Superdispersant 25, F52 = Finasol OSR 52, and SG = Slickgone NS. *** *p* < 0.001, ** *p* < 0.01, and * *p* < 0.05.

Immediately after adding NSO-1 crude oil, the concentration of *n*-alkanes (C_12_ to C_26_) plus branched alkanes (pristane and phytane) was 50.59 (±3.41) µg mL^−1^ (Figure 1) and that of PAHs (mainly naphthalene, anthracene, phenanthrene and their methylated derivatives) was 4.37 (±1.09) µg mL^−1^ (see Supplementary Figure S3 for full oil composition). At day 1, no significant biodegradation of either alkanes or PAHs was observed in the oil-only controls or in microcosms treated with sophorolipid. In contrast, the addition of dispersants and the other two biosurfactants significantly enhanced total alkane biodegradation relative to both the time-zero controls (21–42% reduction; coef. = −0.75, *t* = −3.85, *p* < 0.01) and the oil-only controls (20–38% reduction; coef. = −0.56, *t* = −3.13, *p* < 0.05). There was no significant (*p* > 0.05) reduction in the concentration of any PAHs in any treatments at day 1.

By day 3, the total concentration of alkanes had decreased further; however, there were no significant differences between any treatments, as biodegradation in the oil-only controls and sophorolipid treatments matched those in all other treatments. By day 7, the reduction in total alkane concentration was on average >80%, increasing further to 96–100% by day 14. PAH-biodegradation did not differ significantly throughout the experiment in any treatments, but total concentration reduced on average by 88% by day 7 and by 92% by day 14. All losses are attributed to biodegradation, as no abiotic losses were observed in the killed controls.

### 3.3. Effects of Oil and/or Dispersants/Biosurfactants on Bacterial Community Composition and Abundance

**Figure 2:**
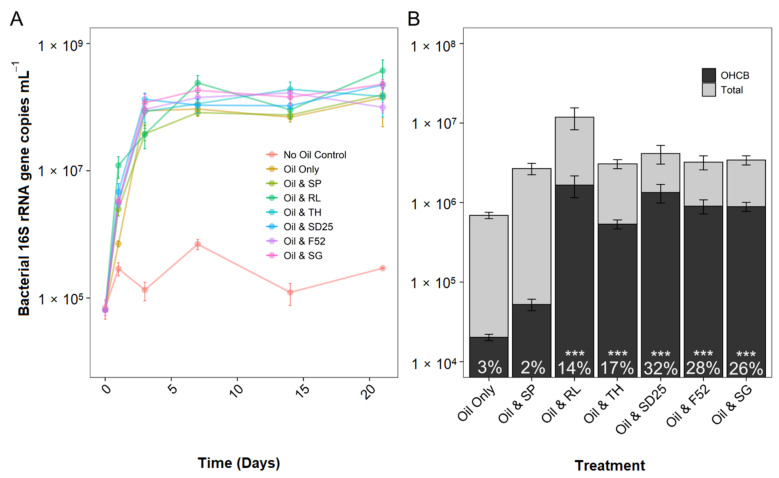
Bacterial 16S rRNA gene abundance (mean ± SE, *n =* 3) over a 21 day period for total bacteria (**A**) and at day 1 only, for total bacteria and proportion (percentage figures) of obligate hydrocarbonoclastic bacteria (**B**). OHCB sequences are defined as from the genera: *Alcanivorax*, *Cycloclasticus*, *Oleibacter*, *Oleispira*, *Thalassolituus*. All microcosms contained seawater, nutrients, and oil (NSO-1 0.1% *v*/*v*), and some also contained additional dispersants or biosurfactants (0.005% *v*/*v*; 20:1 ratio of oil). Dispersant and biosurfactant codes are as follows: SP = sophorolipid, RL = rhamnolipid, TH = trehalolipid, SD25 = Superdispersant 25, F52 = Finasol OSR 52, and SG = Slickgone NS. Asterisks above percentage figures indicate significant differences in mean OHCB relative abundance compared to the oil-only control (*** *p* < 0.001).

To quantify the effects of dispersants and biosurfactants, when applied to oil-contaminated microcosms, on bacterial abundance, diversity, and community composition, bacterial 16S rRNA genes were analysed by qPCR and amplicon library sequencing. High-throughput 16S rRNA sequencing resulted in an average of 12,836 (range 717–177,558) sequence reads per sample for Bacteria. At day 1, the bacterial 16S rRNA gene abundance had significantly increased (coef. = 1.46, *t* = 4.83, *p* < 0.001) in all treatments containing dispersants or biosurfactants (except sophorolipid) in comparison to the oil-only controls (ranging from 4-fold (trehalolipid) to 17-fold (rhamnolipid); Figure 2A,B). Additionally, those dispersants and biosurfactants which significantly reduced the interfacial surface tension between oil and water stimulated a significant increase (14–32%) in the relative abundance of 16S rRNA genes assigned to obligate hydrocarbonoclastic bacteria (OHCB) relative to the oil-only and sophorolipid treatments (which remained in the region of 2–3%) (Figure 2B). Energy and carbon for OHCB comes almost exclusively from hydrocarbons and the OHCB exhibit the “BIOLOG anomaly”, in that they grow on only two of the 95 BIOLOG substrates (Tween 40 and Tween 80) [76]. Whilst some pure cultures of OHCB have been demonstrated to grow on a few non-hydrocarbon substrates [77,78] we refer to them hereafter as OHCB to distinguish between these highly competitive hydrocarbon degraders and those more metabolically diverse bacteria, which potentially can also degrade hydrocarbons. It should be noted that the 16S rRNA phylogenetic distance between ASVs and known OHCB is not a guarantee that those ASVs are themselves OHCB. However, for the purposes of this study, they are assumed so. The increase in OHCB by day 1 resulted in distinct bacterial communities in all dispersant and biosurfactant treatments in comparison to the oil-only controls and sophorolipid treatments (Supplementary Figure S4). From day 3 onwards, bacterial 16S rRNA gene abundance in the oil-only controls increased to levels comparable to those in dispersant and biosurfactant treatments (Figure 2A). Bacterial communities in the oil-only and sophorolipid treatments remained distinct from the other treatments at day 3 until becoming similar by day 7 and thereafter (Supplementary Figure S4). The plateau of bacterial growth at day 3 is strongly correlated (0.86 *R*^2^ to 0.99 *R*^2^, *p* < 0.001) to the nearly complete reduction in ammonia (−99% by day 1). Phosphorus was reduced across all treatments by −53% by day 1 and by −78% by day 21 (Supplementary Figure S5).

**Figure 3:**
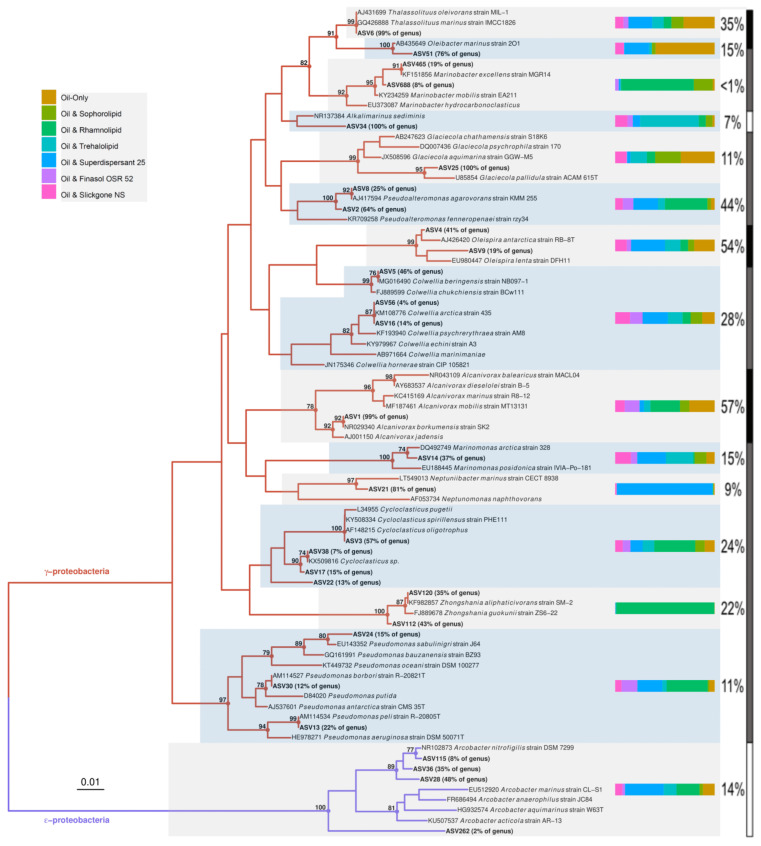
Unrooted Neighbour-Joining phylogeny based on 16S rRNA gene sequences from representative bacterial ASVs which increased in relative abundance in microcosms containing seawater, nutrients, oil (NSO-1 0.1% *v*/*v*), and additional dispersants or biosurfactants (0.005% *v*/*v*; 20:1 ratio to oil) compared with oil-only. ASV sequences are aligned with known hydrocarbon-degrading bacteria and closest relatives; bootstrap values >70 displayed (1000 iterations). Evolutionary distances computed by Maximum Composite Likelihood protocol (using the Tamura-Nei model (Tamura and Nei, 1993)), sum of branch length = 1.42. Analysis involved 85 nucleotide sequences (including 29 ASVs and 66 related strains), with a total of 258 positions in the final dataset. Evolutionary analyses were conducted in MEGA7. Percentage figures in parentheses next to individual ASVs display the relative abundance of that sequence within the assigned genus. Horizontal bars (right of clades) represent the proportion of sequences assigned to ASVs within the clade in the different treatments (see colour key) over all time points (excluding day zero). Percentage figures next to horizontal bars show the maximum relative abundance reached for ASVs within that clade in any treatment at any time point (excluding day zero; see additional figures for treatment-specific bacterial succession). Vertical bars (right of percentage figures) represent OHCB (black), genera where some isolates have grown on hydrocarbons (grey), and genera that have increased in relative abundance in oil-contaminated environments, but no isolates have been shown to degrade hydrocarbons (white).

Increases in the absolute abundance of Bacteria were driven by increases in the relative abundance of particular ASVs. In addition to known OHCB, key ASVs were investigated based on increases in relative abundance across multiple treatments, or selection by specific dispersants or biosurfactants (Figure 3).

**Figure 4:**
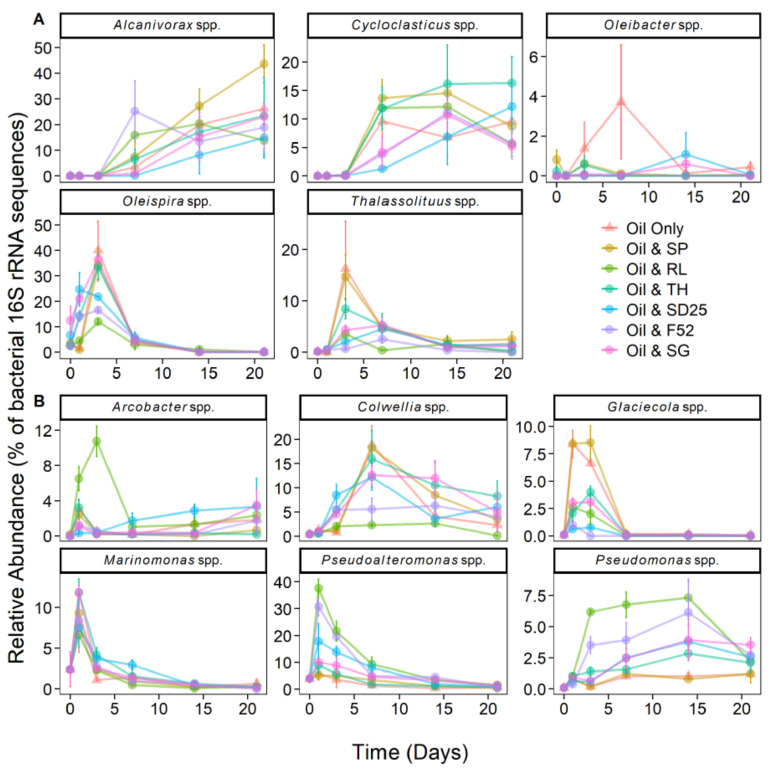
Relative abundance (% of the bacterial community; mean ± SE, *n =* 3) of 16S rRNA gene sequences within ASVs assigned to obligate hydrocarbonoclastic bacteria (**A**), and bacteria that were abundant (and are often associated with hydrocarbon degradation; see Supplementary Table S2) (**B**), over a 21 day period. All microcosms contained seawater, nutrients, and oil (NSO-1 0.1% *v*/*v*), and some also contained additional dispersants or biosurfactants (0.005% *v*/*v*; 20:1 ratio of oil). Dispersant and biosurfactant codes are as follows: SP = sophorolipid, RL = rhamnolipid, TH = trehalolipid, SD25 = Superdispersant 25, and F52 = Finasol OSR 52, SG = Slickgone NS.

*Oleispira* and *Thalassolituus* 16S rRNA gene amplicons increased in relative abundance at day 1 and 3, respectively, followed by increases in *Alcanivorax* and *Cycloclasticus* sequences at day 7 (Figure 4A). This pattern of succession was consistent across all treatments, though the timing of increase was different between treatments (for example, growth of *Oleispira* was less in treatments containing rhamnolipid). OHCB growth developed later in the oil-only controls and sophorolipid treatments, corresponding with delayed alkane biodegradation. At day 1, 16S rRNA gene amplicons from the alkane-degrading genus *Oleispira* (represented by ASV4 and ASV9, Figure 3 and Figure 4A) had significantly increased in relative abundance to 14–25%, from background levels (3%), in microcosms containing Finasol OSR 52, Slickgone NS, Superdispersant 25, and trehalolipid (coef. = 0.14, *t* = 4.23, *p* < 0.05). By day 3, *Oleispira* 16S rRNA gene amplicons had also increased in the oil-only controls (to 40%) and sophorolipid treatment (to 34%) to levels found in all other treatments. 16S rRNA gene amplicons from the alkane-degrading genus *Thalassolituus* (represented by ASV6, Figure 3 and Figure 4A) increased in all treatments by day 3, although the relative abundance was significantly higher in the oil-only controls (16%) and sophorolipid (15%) treatments (coef. = 11.89, *t* = 4.16, *p* < 0.05). 16S rRNA gene amplicons from the alkane-degrading genus *Oleibacter* (represented by ASV51, Figure 3 and Figure 4A) grew in numerous treatments, and though its relative abundance remained comparatively low throughout (0–6%), larger increases were observed in the oil-only controls. By days 7 or 14 (depending on the treatment) and through to day 21, 16S rRNA gene amplicons from the alkane-degrading *Alcanivorax* (represented by ASV1, Figure 3 and Figure 4A) and the PAH-degrading *Cycloclasticus* (represented by ASVs 3, 17, 22, and 38, Figure 3 and Figure 4A) became the most dominant OHCB in all treatments (representing 8–42% and 5–16% at days 14 and 21, respectively).

ASVs assigned to more metabolically versatile bacteria had more between-treatment variability compared to ASVs assigned to OHCB. These genera contain species that have been shown to degrade hydrocarbons, and/or that have been observed to increase in abundance in oil-contaminated marine environments (Supplementary Table S2). However, like many prior studies (Supplementary Table S2) it cannot always be assumed that such genera are degrading hydrocarbons, although large increases in relative abundance may often suggest that this is the case. *Pseudomonas* 16S rRNA gene amplicons (represented by ASVs 13, 24, and 30, Figure 3 and Figure 4B) increased in relative abundance in most treatments by days 1 and 3 and remained above background levels (~0.09%) across all treatments (1–4%) by day 21. Additionally, there was a selection for *Pseudomonas* in Finasol OSR 52, and more so rhamnolipid, treatments, where significant increases in relative abundance (coef. = 0.12, *t* = 4.13, *p* < 0.05) were observed by days 3 (4–6%), 7 (4–7%), and 14 (6–7%). In addition, the patterns of increase in the genera *Arcobacter*, *Colwellia*, *Glaciecola*, *Marinomonas*, and *Pseudoalteromonas* are shown in Figure 4B, their capacity for hydrocarbon degradation referenced in Supplementary Table S2, and their phylogenetic position of representative ASVs in Figure 3. Furthermore, the genera *Alkalimarinus*, *Neptuniibacter* and *Zhongshania* significantly increased in relative abundance in trehalolipid, Superdispersant 25, and rhamnolipid treatments, respectively (Supplementary Figure S6).

The Ecological Index of Hydrocarbon Exposure (EIHE), which quantifies the proportion of the bacterial community with hydrocarbon biodegradation potential [60], significantly increased (coef. = 0.37, *t* = 4.43, *p* < 0.01) at day 1 in oil microcosms containing Superdispersant 25 (0.54), rhamnolipid (0.58), and Finasol OSR 52 (0.59) in comparison to day zero (0.05) and to the oil-only controls (0.15), and sophorolipid treatments (0.14) (Supplementary Figure S7). By day 3, the index measured in the oil-only controls and sophorolipid treatments had increased significantly (0.61–0.68; coef. = 0.48, *t* = 5.87, *p* < 0.001) from day 1. By Day 7, there is a decrease in the EIHE in all treatments apart from those containing rhamnolipid and Superdispersant 25, which demonstrate an increase in the EIHE. By day 21, the index ranged from 0.42 to 0.66 across all treatments, remaining well above the index at day zero (0.05) prior to oil addition.

### 3.4. Effects of Oil and/or Dispersants/Biosurfactants on Bacterial Community Dynamics

**Figure 5:**
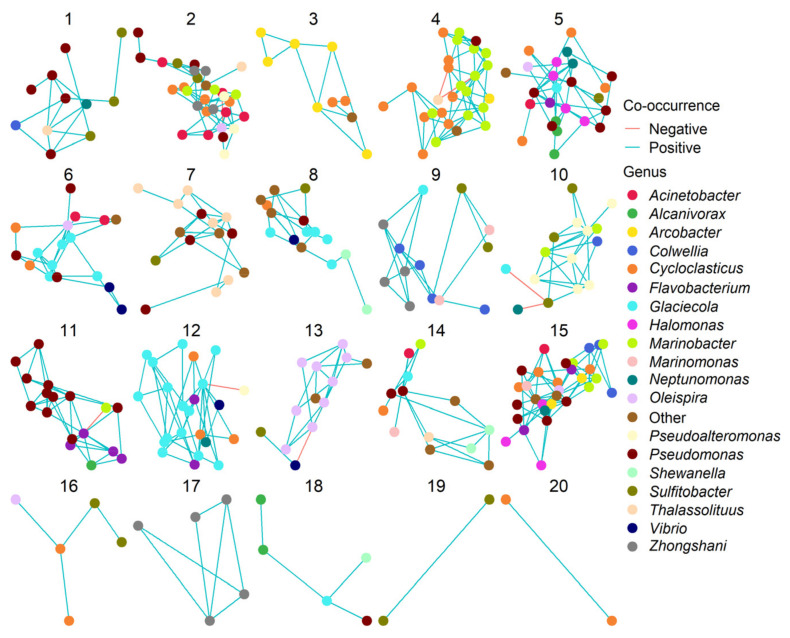
Network modules based on clustering of a SPIEC-EASI co-occurrence network formed of ASVs from bacterial genera associated with hydrocarbon degradation. Each point represents a single ASV, which are coloured according to their identity at the genus level (RDP classifier confidence >0.8). Blue edges represent positive co-occurrences between ASVs across the experiment, whereas red edges show negative co-occurrences.

**Figure 6:**
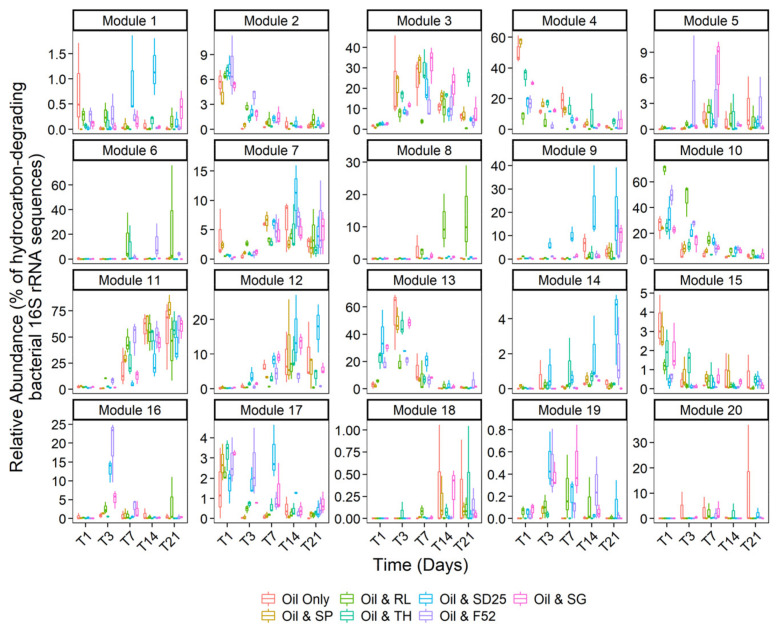
Relative abundances of modules based on clustering of a SPIEC-EASI co-occurrence network of ASVs from bacterial genera associated with hydrocarbon degradation. The total relative abundance (as a proportion of bacterial genera associated with hydrocarbon degradation) of ASVs in each module across the course of the experiment, based on counts of 16S rRNA sequences. All microcosms contained seawater, nutrients, and oil (NSO-1 0.1% *v*/*v*), and some also contained additional dispersants or biosurfactants (0.005% *v*/*v*; 20:1 ratio of oil). Dispersant and biosurfactant codes are as follows: SP = sophorolipid, RL = rhamnolipid, TH = trehalolipid, SD25 = Superdispersant 25, F52 = Finasol OSR 52, and SG = Slickgone NS.

The ASV table was filtered to ASVs confidently identified as belonging to possible hydrocarbon-degrading genera (as defined by the EHIE index [60]). Network construction revealed co-occurrences between taxa that were largely dominated by positive, rather than negative, co-occurrences (Supplementary Figure S8A). ASVs in the network co-occurred with 5.55 others on average, as shown by the network node degree distribution (Supplementary Figure S8B). Clustering of the network revealed 20 modules, with each module consisting of between 2 and 30 ASVs. The network had an overall modularity score 0.37, indicating that ASVs in the network formed sub-communities of co-occurring ASVs. One feature of many of the modules was the dominance of ASVs from specific genera (Figure 5). For example, module 17 was composed entirely of *Vibrio* ASVs that positively co-occurred with each other, whilst modules 3, 7, 11, 12, and 13 had high proportions of *Arcobacter*, *Thalassolituus*, *Pseudomonas*, *Glaciecola*, and *Oleispira* ASVs, respectively. The propensity of ASVs within a genus to co-occur with others from the same genus is referred to as taxonomic assortativity. Our network had a taxonomic assortativity coefficient of 0.21, indicating positive assortativity. We tested the association between an ASV’s genus and its module using a Fisher test, revealing ASVs from different genera were not randomly distributed between modules, further supporting the role of taxonomic assortativity in our network. Examination of the total relative abundance (as a fraction of the hydrocarbon-degrading proportion of the bacterial community) revealed that many modules showed distinct treatment or successional peaks in abundance (Figure 6). Module 10 (47% *Pseudoalteromonas* ASVs), module 13 (69% *Oleispira* ASVs), and module 17 (100% *Vibrio* ASVs) were more abundant early in the experiment, peaking in abundance during days 1 and 3, and for *Vibrio* day 7. In contrast, modules 5 (12% *Alcanivorax* and 24% *Pseudomonas* ASVs) and 11 (24% *Cycloclasticus* and 65% *Pseudomonas* ASVs) peaked later in the experiment, supporting the successional trends observed at the individual genus level. Whilst ASVs within modules were predominantly connected by strong positive co-occurrences with each other, co-occurrences between ASVs from different modules were significantly weaker (Figure S9; coef. = −0.09, *t* = −18.43, *R*^2^ = 0.21, *p* < 0.001). The weaker co-occurrences linking modules provides evidence against the concept of competitive exclusion in oil-degrading communities, where we might expect strongly negative co-occurrences to link species in different successional stages.

## 4. Discussion

### 4.1. To What Extent Do Different Dispersants and Biosurfactants Enhance the Growth of Hydrocarbon-Degrading Bacteria and Accelerate Hydrocarbon Degradation?

After only 24 h, there was a clear selection for hydrocarbon-degrading bacteria (HCB) in all treatments where dispersants or biosurfactants had significantly reduced the interfacial surface tension between oil and water, which was coupled with significant reductions in the concentration of alkanes (21–42%). The significantly enhanced rate of alkane biodegradation occurred under high nutrient concentrations (during the first 24 h), including both ammonium and nitrate, typical of eutrophic marine estuarine environments such as the Thames Estuary and Liverpool Bay [44,79], which continually receive riverine ammonium and nitrate. Similar rapid alkane biodegradation may not be observed in environments with significantly lower total N or in nitrate-dominant systems, such as many open water environments. However, a previous microcosm study of the Thames Estuary found little difference in hydrocarbon degradation rates or bacterial populations between samples from microcosms with similar levels of nitrate alone or amended with nitrate plus ammonium, with the same hydrocarbon-degrading bacteria growing regardless of which nitrogen sources were present [45]. It has also been demonstrated that both ammonium and nitrate support extensive biodegradation of crude oil, with similar rates of hydrocarbon biodegradation [80]. Moreover, whilst hydrocarbon biodegradation may be extremely limited in ultraoligotrophic environments, the use of dispersants could assist hydrocarbon biodegradation by rapidly reducing the concentration, and increasing the surface area, of oil. This happens because dispersants transform oil on the surface of the water into small droplets (10–300 μm, [81,82,83]) that become dispersed into the underlying water column. This dispersion of oil provides an increased surface area for microbial attachment, and biodegradation will proceed at lower nutrient concentrations as oil is dispersed over a wider area [7]. Additionally, an increased oil surface area to volume ratio allows hydrocarbon-degrading microbes to expend more energy on growth and less energy producing biosurfactants, thereby accelerating biodegradation [13,84]. However, Kleindienst et al. [20] demonstrated that the use of dispersants allowed the hydrocarbon- and dispersant-degrading genus *Colwellia* to outcompete certain HCB (*Marinobacter*), and thus provided no hydrocarbon biodegradation advantage. Conclusions reached by Kleindienst et al. [20] were likely due to differences in experimental procedure, as well as differences in indigenous bacterial communities between the Gulf of Mexico and the North Sea. Kleindienst et al. [20] used water accommodated fractions (WAFs) (sampled seawater was stored for >1 month), whereby oil was mechanically and chemically dispersed with Corexit 9500. Kleindienst et al. [20] adopted this method as they wanted to replicate the *Deepwater* oil plume, which consisted of the water-accommodated fraction, after the *Deepwater* Horizon well-blowout. Additionally, sampling of WAFs first occurred after seven days. Our study aimed to replicate the application of dispersants to the water’s surface. By contrast, dispersants and biosurfactants, which were different from Corexit, were added directly to the seawater surface, avoiding vigorous mechanical dispersion. Additionally, microcosms, which were set up immediately after seawater sampling, were sampled at day 1, providing evidence of increased hydrocarbon degradation with the application of dispersants and biosurfactants in the very early stages (except when sophorolipid was used).

By day 3, alkane biodegradation and abundance of HCB in the oil-only controls and sophorolipid treatments had caught up with, and was equal to, all other treatments. This finding could be due partly to inorganic nitrogen becoming limited within the first few days and suggests that the later blooms of *Alcanivorax* and *Cycloclasticus* after 7 days may rely on the turnover of organic nitrogen as cells die. As with the alkanes, PAHs were biodegraded, but there were no significant differences between any treatments. HCB able to degrade *n*-alkanes are known to grow more rapidly than those degrading branched alkanes and PAHs [12,85], during which time, in this study, nutrients had been depleted. Had there been a constant supply of inorganic nutrients, as expected during oil dispersed within some marine environment, microcosms containing dispersed oil may have maintained their enhanced biodegradation for longer, which could possibly also translate into significantly enhanced PAH reduction.

Dispersal and dilution of oil within microcosms is limited due to their confined nature, in contrast to oil spills in open waters, where oil can be diluted rapidly to sub-ppm concentrations [86]. Dispersion over a wide area and lower concentration of oils may allow access to further inorganic nutrients and therefore lead to faster degradation of dispersed oil compared to undispersed oil on the sea surface. It has also been highlighted that ex-situ bottle experiments allow for higher oil concentrations which can, in some cases, inhibit hydrocarbon degradation [15,87]. Furthermore, Nedwed and Coolbaugh [88] state that microcosm biodegradation experiments can be negatively biased due to the physical constraints of the container limiting the spread of oil. However, despite these previous findings, this study has demonstrated that even with a relatively high oil concentration, and the confined nature of microcosms, both dispersants and biosurfactants significantly increased the rate of hydrocarbon biodegradation and the abundance of hydrocarbon-degrading bacteria.

This study has provided evidence that the biosurfactants rhamnolipid and trehalolipid have the same ability to enhance hydrocarbon degradation as commercial dispersants. However, their potential as a replacement for dispersants is currently limited due to the exceptionally high costs of large-scale production [89,90]. Sophorolipid reduced surface tension of the seawater to a much lesser extent than all other surfactants or dispersants, which could suggest why there was no significant increase in hydrocarbon biodegradation, like the oil-only control. In contrast, sophorolipid has previously been observed to increase hydrocarbon degradation in oiled soil slurry reactors [91,92]. Sophorolipid has been noted to decrease the surface tension of water from ~70 to ~36 mN m^−1^, though this relies on a minimum sophorolipid concentration of 15–20 ppm [93,94]. In this study, the final concentration of sophorolipid was 7.5 ppm, and seawater surface tension (without oil) only decreased from 70 to ~60 mN m^−1^. All dispersants and biosurfactants were added at 0.005% *v*/*v* oil to dispersant, replicating industry standard [46], suggesting that an increased initial sophorolipid concentration, or decreased ratio of oil to biosurfactant, would be required for sophorolipid to be effective.

### 4.2. The Psychrophilic OHCB Genus Oleispira Dominates in the First Few Days at the Relatively High Temperature of 16 °C

The initial relative abundance of OHCB in the seawater was very low, with only ASVs from the genus *Oleispira* detectable, at approximately 3% (measured at “time-zero”). ASV4 had a 98.97% 16S rRNA sequence identity to the type strain isolated from Antarctica (closest match to *O. antarctica* strain RB-8T; Figure 3), which in pure culture grows well at 16 °C (the same temperature at which the microcosms were incubated), which is slightly beyond its broad growth optimum of 1–15 °C [95]. *Oleispira* spp. bloom in cold waters (5 °C) but are outcompeted by other OHCB at warmer temperatures [45,96,97].

The sampled seawater was measured at 13.8 °C and therefore not the most suited environment for the psychrophilic *Oleispira*, which may suggest why, despite being the first OHCB to respond to the presence of oil, it was rapidly outcompeted. However, the relatively high abundance of *Oleispira in situ* would provide a competitive advantage (i.e., a priority effect), allowing it to become the dominant OHCB by day 1, constituting 14–25% of sequence reads in four treatments (with lower abundance (1–4%) in oil-only, sophorolipid, and rhamnolipid microcosms). Thereafter, *Oleispira* is outcompeted by OHCB that are more competitive at 16 °C. *O. antarctica* has previously been observed to grow rapidly within 1 day in microcosms with North Sea water and oil (0.01% *v*/*v* which is the same concentration used in this study) [98], though these microcosms were incubated at 4 °C. Alternatively, the dominance of the *Oleispira* ASVs in the warmer microcosms (16 °C) of this study could potentially be due to ASV4 (which accounted for 96% of the relative abundance of sequences assigned to the *Oleispira* genus on days 1 and 3) having a more mesophilic phenotype than currently cultivated *Oleispira* species. This would also help explain its relatively high (1–3%) abundance within the in situ 13.8 °C seawater and suggest a wider diversity within the genus than is currently known. Then, again, many studies have evaluated HCB over a broader time series [20,99,100], whereas in this study a finer timeline was adopted (1 and 3 days). This finer timeline exposed the ability of *Oleispira* spp. to initially dominate in response to oil contamination prior to being rapidly outcompeted, whereas this result may have gone undetected in studies of broader timelines.

### 4.3. Does Niche Partitioning Explain the Observed OHCB Succession?

By day 1, ASVs from the alkane-degrading genus *Oleispira* had proliferated in many treatments. However, ASVs from the genera *Marinomonas* and *Pseudoalteromonas* had also increased in relative abundance. This is likely due to the fact *Marinomonas* and *Pseudoalteromonas* are known PAH degraders (Supplementary Table S2). *Pseudoalteromonas*, and genera such as *Colwellia* and *Cycloclasticus* (which grew in this study by days 3 and 7, respectively), proliferated in the aromatic hydrocarbon-rich oil plume after the *Deepwater* Horizon well-blowout [12]. By day 3, ASVs from the genus *Oleispira* maintained a high relative abundance in the Finasol OSR 52, Slickgone NS, Superdispersant 25, and trehalolipid treatments. However, by day 3, *Oleispira* also grew significantly in the oil-only controls, sophorolipid, and rhamnolipid treatments where it had initially been outcompeted by *Glaciecola* spp. or *Arcobacter* spp. Growth of *Oleispira* was also coupled with increased relative abundance of the alkane-degrading OHCB genus *Thalassolituus* (Figure 3) [101], potentially suggesting niche partitioning by the degradation of different alkanes. For example, *Oleispira antarctica* has been shown to grow only on alkanes up to chain length of *n*-C_24_ [95], but *Thalassolituus oleivorans* can grow on alkanes up to *n*-C_32_ due to the possession of subterminal Baeyer-Villiger monooxygenase alkane oxidation pathways for longer-chained alkanes [102]. By day 7, a significant increase is observed in relative abundance of ASV1 (alkane-degrading genus *Alcanivorax* [102,103]; 99.67% 16S rRNA sequence identity match to *A. borkumensis* SK2; Figure 3), prior to becoming dominant by days 14 and 21. The dominance of *Alcanivorax* occurs when shorter *n*-alkanes and inorganic nitrogen are mostly depleted. This is likely due to the ability of *Alcanivorax* to use both long-chained alkanes and branched alkanes (pristane and phytane) [104] as well as specific systems for scavenging nutrients in oligotrophic environments [105]. Four ASVs from the PAH-degrading genus *Cycloclasticus* (Figure 3) [106], which also has specific systems for scavenging nutrients in oligotrophic environments [107], increased in relative abundance across all treatments by day 7, as alkanes are often degraded prior to more complex PAH molecules. Due to this, *Alcanivorax* spp. often flourish before *Cycloclasticus* spp. [108,109], though they can co-occur [110], including with the addition of the biosurfactant rhamnolipid [111]. This co-occurrence was observed in this study (Modules 5 and 11, Figure 5), from day 7 onwards, and is likely due to the fact they do not compete for the same substrates. Additionally, it has been documented that *Alcanivorax* spp. may enhance PAH degradation [111], potentially due to their biosurfactant enhancing PAH bioavailability. The six dispersants and biosurfactants, with their different compositions, had no significant effect on OHCB succession, with similar patterns observed across all treatments.

### 4.4. Do Dispersants or Biosurfactants Select for Specific Bacterial Genera?

Specific biosurfactant or dispersant treatments resulted in selection for certain genera, which were either undetected or had significantly lower relative abundance in the other treatments. This occurred in treatments containing trehalolipid, Superdispersant 25, and rhamnolipid. The biosurfactant trehalolipid triggered a significant increase in the relative abundance of ASV34, from the genus *Alkalimarinus* (97.29% 16S rRNA sequence identity to *A. sediminis*; Figure 3) after 3 days. *Alkalimarinus* spp. are not known hydrocarbon degraders. However, recently, *A. sediminis* was found to significantly increase in relative abundance when PAHs bioaccumulated in bivalves [112]. Superdispersant 25 resulted in a significant increase in the relative abundance of ASV21 from the genus *Neptuniibacter* (97.34% 16S rRNA sequence identity to *N. marinus* strain CECT 8938; Figure 3) after 3 days. The selection of *Neptuniibacter*, some strains of which contain the *soxA-D* gene cluster for the oxidization of sulphur [113], could be due to Superdispersant 25 containing sulphur [114]. The biosurfactant rhamnolipid stimulated a significant increase in the relative abundance of two ASVs from the genus *Zhongshania* (98.34% and 96.35% 16S rRNA sequence identity to *Z. aliphaticivorans* strain SM-2; a known alkane-degrader [115] by day 21. *Zhongshania* sp. has been observed as an abundant alkane-degrader in Norwegian seawater [116].

### 4.5. Rhamnolipid Stimulates Pseudomonas Dominance—A Case of Advantageous Compatibility with Its Own Biosurfactant?

During the first 3 days, rhamnolipid selected for four ASVs from the genus *Arcobacter* and two ASVs from the genus *Pseudoalteromonas* (Figure 3). *Arcobacter* has been observed to increase in abundance in oiled environments (Supplementary Materials Table S2), especially oil sands [117], whilst some strains of *Pseudoalteromonas* spp. have been shown to grow on both *n*-alkanes and PAHs [118]. However, by day 7, ASVs from the genera *Arcobacter* and *Pseudoalteromonas* decrease in relative abundance as ASVs from the genus *Pseudomonas* (Figure 3) were observed to significantly increase. This selection for *Pseudomonas* by rhamnolipid can be observed in module 11 (Figure 5) of the network analysis, where a bacterial consortium is selected by the rhamnolipid treatment, including *Pseudomonas* ASVs (ASVs 13, 24, and 30) which are observed to positively co-occur with others ASVs (e.g., *Alcanivorax* and *Cycloclasticus*).

Certain *Pseudomonas* spp. are known hydrocarbon degraders, including the PAH naphthalene [119] and *n*-alkanes ranging from C_8_-C_36_ [120]. *P. aeruginosa* is known as a prominent producer of the biosurfactant rhamnolipid in the presence of hydrocarbons [121], though some other species can also produce this class of glycolipid, including *P. chlororaphis*, *P. plantarii*, *P. putida*, and *P. fluorescens* [24,122]. Moreover, specific *Pseudomonas* spp. are known to increase the rate of hydrocarbon degradation in the presence of rhamnolipid addition [123]. The significant increase in relative abundance of *Pseudomonas* spp. in microcosms containing rhamnolipid is potentially a form of advantageous compatibility. Rhamnolipids, whilst increasing hydrocarbon bioavailability, also modulate the swarming motility of *Pseudomonas* spp. [124,125]. Hydrophobic compounds (i.e., hydrocarbons) are often surface-associated, and chemotaxis, via swarming, enhances their bioavailability/biodegradation [126,127,128]. Furthermore, rhamnolipid is a known antimicrobial [129], therefore where the same biosurfactant is used to swarm over the hydrocarbon surface, this may prevent the establishment of competing microbes [130], providing a competitive advantage over other hydrocarbon degraders in rhamnolipid treatments.

## 5. Conclusions

Overall, this study has highlighted that dispersants and biosurfactants (excluding sophorolipid) not only reduce the interfacial surface tension of oil and water but also significantly increase the abundance of HCB, and thus the rate of hydrocarbon biodegradation over the first 24 h. Moreover, this study provides evidence that niche partitioning drives a succession of obligate hydrocarbonoclastic bacteria (OHCB), and that the OHCB *Oleispira*, hitherto considered to be a psychrophile, can dominate in the early stages of oil-spill response (the first 3 days), out-competing other OHCB, even at 16 °C. Additionally, some dispersants or biosurfactants select for specific bacterial genera, especially the biosurfactant rhamnolipid, which appears to provide an advantageous compatibility with the genus *Pseudomonas*. However, this study was carried out in microcosms with an initial abundance of nutrients (first 24 h) which then quickly became limiting (day 3 onwards). Given the variability between many marine environments (e.g., nutrient concentrations, wave/wind energy, temperature) and oil spills (e.g., oil concentration, oil type, oil:dispersant ratio), caution must be exercised when extrapolating results of such studies to different field scenarios such as low-nutrient open water systems. Therefore, further research into the efficiency of dispersant/biosurfactant application to oil slicks in a wide range of environmental conditions is required.

## Data Availability

Raw sequence data have been submitted to the European Nucleotide Archive database under accession number PRJEB37243.

## Supplementary Materials

The following are available online at https://www.mdpi.com/article/10.3390/microorganisms9061200/s1. Figure S1: Map of sampling site, United Kingdom (A); red circle indicates location for sampling site, which is located at Earls Hope salt marsh, Stanford le Hope, Thames Estuary, Essex (B). Figure S2: Surface tension (mean ± SE, *n =* 3) measured in microcosms containing seawater or seawater with additional dispersants or biosurfactants (0.005% *v*/*v*). Dispersant and biosurfactant codes are as follows: SP = sophorolipid, RL = rhamnolipid, TH = trehalolipid, SD25 = Superdispersant 25, F52 = Finasol OSR 52, and SG = Slickgone NS. Stars (*** *p* < 0.001) and octothorpes (### *p* < 0.001) indicate significant changes in mean surface tension compared to seawater and sophorolipid, respectively. Figure S3: Initial (time zero) measured hydrocarbon concentrations (mean ± SE, *n =* 3) within microcosms, including *n*-alkanes (C_12_ to C_26_), branched alkanes pristane and phytane, and PAHs (naphthalene and phenanthrene/anthracene (Phen/Anth) and their methylated derivatives). Figure S4: NMDS (non-metric multidimensional scaling) ordination, based on clustered bacterial 16S rRNA ASVs at a 97% similarity threshold, displaying the effect of treatment, over time, on microbial community composition. Surfactant codes as follows: SP = sophorolipid, RL = rhamnolipid, TH = trehalolipid, SD25 = Superdispersant 25, F52 = Finasol OSR 52, and SG = Slickgone NS. Figure S5: Ammonium (A) and phosphate (B) (mean ± SE, *n =* 3) in microcosms containing seawater, nutrients, and oil (NSO-1 0.01% *v*/*v*), and those with additional dispersants or biosurfactants (0.005% *v*/*v*). Dispersant and biosurfactant codes are as follows: SP = sophorolipid, RL = rhamnolipid, TH = trehalolipid, SD25 = Superdispersant 25, F52 = Finasol OSR 52, and SG = Slickgone NS. Figure S6: Relative abundance (% of the bacterial community; mean ± SE, *n =* 3) of 16S rRNA gene sequences within ASVs assigned to Bacteria associated with oil-degradation (see Supplementary Table S2) over a 21 day period. All microcosms contained seawater, nutrients, and oil (NSO-1 0.1% *v*/*v*), and some also contained additional dispersants or biosurfactants (0.005% *v*/*v*; 20:1 ratio of oil). Dispersant and biosurfactant codes are as follows: SP = sophorolipid, RL = rhamnolipid, TH = trehalolipid, SD25 = Superdispersant 25, F52 = Finasol OSR 52, and SG = Slickgone NS. Figure S7: Mean Ecological Index of Hydrocarbon Exposure (ratio %) representing relative abundance of oil-degrading/associated Bacteria, Lozada et al., 2014) measurements (± SE, *n =* 3) in microcosms containing seawater, nutrients, and oil (NSO-1 0. 1% *v*/*v*), and those with additional dispersants or biosurfactants (0.005% *v*/*v*). Dispersant and biosurfactant codes are as follows: SP = sophorolipid, RL = rhamnolipid, TH = trehalolipid, SD25 = Superdispersant 25, F52 = Finasol OSR 52, and SG = Slickgone NS. Figure S8: (A) edge weight and (B) node degree distributions of the SPIEC-EASI co-occurrence network based on ASVs from bacterial genera associated with hydrocarbon degradation. Edge weights indicate the direction and effect size of co-occurrences between ASVs, whilst node degree values indicate the number of ASVs each ASV co-occurs (positively or negatively) with. In (A), the dashed red line indicates 0 (no co-occurrence), whereas in (B) it indicates the median node degree (5.55) of ASVs in the network. Figure S9: Comparison of edge weights connecting ASVs within and between modules. Positive values indicate positive co-occurrences and vice versa, with values closer to 0 indicating weaker co-occurrences. Co-occurrences between ASVs within modules were significantly stronger and more positive than those between modules (coef. = 0.09, *t* = 18.43, *p* < 0.001). Table S1: Experimental design showing 10 treatments (codes in parentheses), including concentrations and measurements of seawater, nutrients, crude oil (NSO-1), dispersant or surfactants, and, for the killed controls, mercuric chloride (HgCl_2_). Table S2: Evidence demonstrating hydrocarbon degradation (alkane of PAH) and growth in oil-polluted marine environments for certain genera of Bacteria (either so-called “obligate hydrocarbonoclastic Bacteria” or more metabolically versatile).

## References

[1] Burger J. (1997). Oil Spills.

[2] Rystad Energy (2020). Global Oil and Gas Discoveries Reach Four-Year High in 2019, Boosted by ExxonMobil’s Guyana Success. https://www.rystadenergy.com/newsevents/news/press-releases/global-oil-and-gas-discoveries-reach-four-year-high-in-2019/.

[3] U.S. Energy Information Administration (2020). Short-Term Energy Outlook (STEO).

[4] ITOPF (2011). Use of Dispersants to Treat Oil Spills.

[5] Gopalan B., Katz J. (2010). Turbulent shearing of crude oil mixed with dispersants generates long microthreads and microdroplets. Phys. Rev. Lett..

[6] Fingas M. (2011). Oil Spill Dispersants: A Technical Summary. Oil Spill Science and Technology.

[7] Prince R.C., McFarlin K.M., Butler J.D., Febbo E.J., Wang F.C.Y., Nedwed T. (2013). The primary biodegradation of dispersed crude oil in the sea. Chemosphere.

[8] Kujawinski E.B., Kido Soule M.C., Valentine D.L., Boysen A.K., Longnecker K., Redmond M.C. (2011). Fate of dispersants associated with the Deepwater Horizon oil spill. Environ. Sci. Technol..

[9] Brandvik P.J., Johansen Ø., Davies E.J., Leirvik F., Krause D.F., Daling P.S., Dunnebier D., Masutani S., Nagamine I., Storey C. (2017). Subsea Dispersant Injection (SSDI)—Summary Findings from a Multi-Year Research and Development Industry Initiative. Int. Oil Spill Conf. Proc..

[10] Atlas R.M., Hazen T.C. (2011). Oil biodegradation and bioremediation: A tale of the two worst spills in U.S. history. Environ. Sci. Technol..

[11] Hazen T.C., Dubinsky E.A., DeSantis T.Z., Andersen G.L., Piceno Y.M., Singh N., Jansson J.K., Probst A., Borglin S.E., Fortney J.L. (2010). Deep-sea oil plume enriches indigenous oil-degrading bacteria. Science.

[12] Dubinsky E.A., Conrad M.E., Chakraborty R., Bill M., Borglin S.E., Hollibaugh J.T., Mason O.U.M., Piceno Y., Reid F.C., Stringfellow W.T. (2013). Succession of hydrocarbon-degrading bacteria in the aftermath of the deepwater horizon oil spill in the gulf of Mexico. Environ. Sci. Technol..

[13] Brakstad O.G., Ribicic D., Winkler A., Netzer R. (2018). Biodegradation of dispersed oil in seawater is not inhibited by a commercial oil spill dispersant. Mar. Pollut. Bull..

[14] Ribicic D., Netzer R., Winkler A., Brakstad O.G. (2018). Microbial communities in seawater from an Arctic and a temperate Norwegian fjord and their potentials for biodegradation of chemically dispersed oil at low seawater temperatures. Mar. Pollut. Bull..

[15] Prince R.C., Coolbaugh T.S., Parkerton T.F. (2016). Oil dispersants do facilitate biodegradation of spilled oil. Proc. Natl. Acad. Sci. USA.

[16] Brakstad O.G., Nordtug T., Throne-Holst M. (2015). Biodegradation of dispersed Macondo oil in seawater at low temperature and different oil droplet sizes. Mar. Pollut. Bull..

[17] Lindstrom J.E., Braddock J.F. (2002). Biodegradation of petroleum hydrocarbons at low temperature in the presence of the dispersant Corexit 9500. Mar. Pollut. Bull..

[18] Rahsepar S., Smit M.P.J., Murk A.J., Rijnaarts H.H.M., Langenhoff A.A.M. (2016). Chemical dispersants: Oil biodegradation friend or foe?. Mar. Pollut. Bull..

[19] Hamdan L.J., Fulmer P.A. (2011). Effects of COREXIT® EC9500A on bacteria from a beach oiled by the Deepwater Horizon spill. Aquat. Microb. Ecol..

[20] Kleindienst S., Seidel M., Ziervogel K., Grim S., Loftis K., Harrison S., Malkin S.Y., Perkins M.J., Field J., Sogin M.L. (2015). Chemical dispersants can suppress the activity of natural oil-degrading microorganisms. Proc. Natl. Acad. Sci. USA.

[21] IPIECA, IOGP (2017). API Guidelines on Implementing Spill Impact Mitigation Assessment, SIMA.

[22] Kirby M., Neall P., Rooke J., Yardley H. (2011). Formulation Changes in Oil Spill Dispersants: Are They Toxicologically Significant?. Oil Spill Science and Technology.

[23] European Maritime Safety Agency (2009). Manual on the Applicability of Oil Spill Dispersants—Version 2.

[24] Chong H., Li Q. (2017). Microbial production of rhamnolipids: Opportunities, challenges and strategies. Microb. Cell Fact..

[25] Banat I.M., Makkar R.S., Cameotra S.S. (2000). Potential commercial applications of microbial surfactants. Appl. Microbiol. Biotechnol..

[26] Kapellos G.E. (2017). Microbial Strategies for Oil Biodegradation. Model. Microscale Transp. Biol. Process..

[27] Singh P., Patil Y., Rale V. (2019). Biosurfactant production: Emerging trends and promising strategies. J. Appl. Microbiol..

[28] Lang S., Wullbrandt D. (1999). Rhamnose lipids—Biosynthesis, microbial production and application potential. Appl. Microbiol. Biotechnol..

[29] Davila A.M., Marchal R., Vandecasteele J.P. (1994). Sophorose lipid production from lipidic precursors: Predictive evaluation of industrial substrates. J. Ind. Microbiol..

[30] White D.A., Hird L.C., Ali S.T. (2013). Production and characterization of a trehalolipid biosurfactant produced by the novel marine bacterium Rhodococcus sp., strain PML026. J. Appl. Microbiol..

[31] Banat I.M. (1995). Biosurfactants production and possible uses in microbial enhanced oil recovery and oil pollution remediation: A review. Bioresour. Technol..

[32] Vijayakumar S., Saravanan V. (2015). Biosurfactants-types, sources and applications. Res. J. Microbiol..

[33] Total-Fluides (2012). Safety Data Sheet Finasol OSR 52.

[34] Oil-Slick-Dispersants (2015). Safety Data Sheet Superdispersant 25.

[35] DASIC-International (2002). Slickgone NS Data Safety Sheet.

[36] Sambles C.M., White D.A. (2015). Genome sequence of Rhodococcus sp. strain PML026, a trehalolipid biosurfactant producer and biodegrader of oil and alkanes. Genome Announc..

[37] ITOPF (2019). Oil Tanker Spill Statistics 2019.

[38] Little D.I., Little A.E. (1991). Estuarine oil spill effects in the context of dispersant use changes. Int. Oil Spill Conf. Proc..

[39] Gilson D. (2006). Report on the Non-Mechanical Response for the T/V Exxon Valdez Oil Spill. https://www.pwsrcac.org/wp-content/uploads/filebase/programs/oil_spill_response_operations/Report%20on%20the%20Non-Mechanical%20Response%20for%20the%20Exxon%20Valdez%20Oil%20Spill.pdf.

[40] Coolbaugh T., Hague E., Cox R., Varghese G. (2017). Joint Industry Sponsored Effort to Evaluate Post-Macondo Dispersant Research. Int. Oil Spill Conf. Proc..

[41] Floch Le S., Dussauze M., François-Xavier M., Claireaux G., Theron M., Le Maire P., Nicolas-Kopec A. (2014). DISCOBIOL: Assessment of the Impact of Dispersant Use for Oil Spill Response in Coastal or Estuarine Areas. Int. Oil Spill Conf. Proc..

[42] Gregson B.H., Metodieva G., Metodiev M.V., Golyshin P.N., McKew B.A. (2018). Differential Protein Expression During Growth on Medium Versus Long-Chain Alkanes in the Obligate Marine Hydrocarbon-Degrading Bacterium *Thalassolituus* oleivorans MIL-1. Front. Microbiol..

[43] Al-Mallah M., Goutx M., Mille G., Bertrand J.C. (1990). Production of emulsifying agents during growth of a marine Alteromonas in sea water with eicosane as carbon source, a solid hydrocarbon. Oil Chem. Pollut..

[44] Greenwood N., Devlin M.J., Best M., Fronkova L., Graves C., Milligan A., Barry J., van Leeuwen S. (2019). Utilising eutrophication assessment directives from freshwater to marine systems in the Thames Estuary and Liverpool Bay, UK. Front. Mar. Sci..

[45] Coulon F., McKew B.A., Osborn A.M., McGenity T.J., Timmis K.N. (2007). Effects of temperature and biostimulation on oil-degrading microbial communities in temperate estuarine waters. Environ. Microbiol..

[46] Fingas M. (2000). The Basics of Oil Spill Cleanup.

[47] Lecomte Du Noüy P. (1919). A new apparatus for measuring surface tension. J. Gen. Physiol..

[48] Klindworth A., Pruesse E., Schweer T., Peplies J., Quast C., Horn M., Glöckner F.O. (2013). Evaluation of general 16S ribosomal RNA gene PCR primers for classical and next-generation sequencing-based diversity studies. Nucleic Acids Res..

[49] McKew B.A., Smith C.J., McGenity T.J., Timmis K.N., Fernandez B.N. (2015). Real-Time PCR Approaches for Analysis of Hydrocarbon-Degrading Bacterial Communities. Hydrocarbon and Lipid Microbiology Protocols.

[50] R Development Core Team (2011). R: A Language and Environment for Statistical Computing.

[51] Callahan B.J., McMurdie P.J., Rosen M.J., Han A.W., Johnson A.J.A., Holmes S.P. (2016). DADA2: High-resolution sample inference from Illumina amplicon data. Nat. Methods.

[52] Wang Q., Garrity G.M., Tiedje J.M., Cole J.R. (2007). Naïve Bayesian classifier for rapid assignment of rRNA sequences into the new bacterial taxonomy. Appl. Environ. Microbiol..

[53] Nei M., Saitou N. (1987). The neighbor-joining method: A new method for reconstructing phylogenetic trees. Mol. Biol. Evol..

[54] Edgar R.C. (2004). MUSCLE: Multiple sequence alignment with high accuracy and high throughput. Nucleic Acids Res..

[55] Felsenstein J. (1985). Confidence Limits on Phylogenies: An Approach Using the Bootstrap. Evolution.

[56] Tamura K., Nei M. (1993). Estimation of the number of nucleotide substitutions in the control region of mitochondrial DNA in humans and chimpanzees. Mol. Biol. Evol..

[57] Wang W., Wang L., Shao Z. (2010). Diversity and Abundance of Oil-Degrading Bacteria and Alkane Hydroxylase (alkB) Genes in the Subtropical Seawater of Xiamen Island. Microb. Ecol..

[58] Alzarhani A.K., Clark D.R., Underwood G.J.C., Ford H., Cotton T.E.A., Dumbrell A.J. (2019). Are drivers of root-associated fungal community structure context specific?. ISME J..

[59] Venables W.N., Ripley B.D. (2002). Modern Applied Statistics with S.

[60] Lozada M., Marcos M.S., Commendatore M.G., Gil M.N., Dionisi H.M. (2014). The Bacterial Community Structure of Hydrocarbon-Polluted Marine Environments as the Basis for the Definition of an Ecological Index of Hydrocarbon Exposure. Microbes Environ..

[61] Clark D.R. ecolFudge. https://github.com/Dave-Clark/ecolFudge.

[62] Kurtz Z.D., Müller C.L., Miraldi E.R., Littman D.R., Blaser M.J., Bonneau R.A. (2015). Sparse and Compositionally Robust Inference of Microbial Ecological Networks. PLoS Comput. Biol..

[63] Becker R.A., Wilks A.R., Brownrigg R., Minka T.P., Deckmyn A. (2016). Package “Maps”: Draw Geographical *Maps*; R Package Version 2.3-6. https://CRAN.R-project.org/package=maps.

[64] Kassambara A. (2020). ‘ggpubr’: “ggplot2” Based Publication Ready Plots; R Package Version 0.3.0. https://CRAN.R-project.org/package=ggpubr.

[65] Oksanen J., Blanchet F.G., Friendly M., Kindt R., Legendre P., McGlinn D., Minchin P.R., O’Hara R.B., Simpson G.L., Solymos P. (2019). Vegan: Community Ecology Package; R Package Version 2.5.6. https://CRAN.R-project.org/package=vegan.

[66] Auguie B. gridExtra: Functions in Grid Graphics; R Package Version 2.3. CRAN Project 2017. https://CRAN.R-project.org/package=gridextra.

[67] Csardi G., Nepusz T. (2006). The igraph software package for complex network research. InterJournal Complex Syst..

[68] Pedersen T.L. (2020). ggraph: An Implementation of Grammar of Graphics for Graphs and Networks; R Package Version 2.0.3. https://CRAN.R-project.org/package=ggraph.

[69] Wilke C.O. (2020). ggtext: Improved Text Rendering Support for “ggplot2”; R Package Version 0.1.0. https://CRAN.R-project.org/package=ggtext.

[70] Hvitfeldt E. (2020). paletteer: Comprehensive Collection of Color Palettes; R Package Version 1.2.0. https://CRAN.R-project.org/package=paletteer.

[71] Lenth R. emmeans: Estimated Marginal Means, aka Least-Squares Means; R Package Version 1.4.7. https://CRAN.R-project.org/package=emmeans.

[72] Hope R.M. (2013). Rmisc: Ryan Miscellaneous. https://CRAN.R-project.org/package=rmisc.

[73] Wilke C.O. Cowplot: Streamlined Plot Theme and Plot Annotations for ggplot2. R Package Version 0.5.0. https://CRAN.R-project.org/package=cowplot.

[74] Bodenhofer U., Kothmeier A., Hochreiter S. (2011). Apcluster: An R package for affinity propagation clustering. Bioinformatics.

[75] Pedersen T.L. Patchwork: The Composer of Plots. Cran 2019. https://CRAN.R-project.org/package=patchwork.

[76] Yakimov M.M., Timmis K.N., Golyshin P.N. (2007). Obligate oil-degrading marine bacteria. Curr. Opin. Biotechnol..

[77] Radwan S.S., Khanafer M.M., Al-Awadhi H.A. (2019). Ability of the so-called obligate hydrocarbonoclastic bacteria to utilize nonhydrocarbon substrates thus enhancing their activities despite their misleading name. BMC Microbiol..

[78] Zadjelovic V., Chhun A., Quareshy M., Silvano E., Hernandez-Fernaud J.R., Aguilo-Ferretjans M.M., Bosch R., Dorador C., Gibson M.I., Christie-Oleza J.A. (2020). Beyond oil degradation: Enzymatic potential of Alcanivorax to degrade natural and synthetic polyesters. Environ. Microbiol..

[79] Maier G., Nimmo-Smith R.J., Glegg G.A., Tappin A.D., Worsfold P.J. (2009). Estuarine eutrophication in the UK: Current incidence and future trends. Aquat. Conserv. Mar. Freshw. Ecosyst..

[80] Wrenn B.A., Haines J.R., Venosa A.D., Kadkhodayan M., Suidan M.T. (1994). Effects of nitrogen source on crude oil biodegradation. J. Ind. Microbiol..

[81] Li Z., Lee K., King T., Boufadel M.C., Venosa A.D. (2008). Assessment of chemical dispersant effectiveness in a wave tank under regular non-breaking and breaking wave conditions. Mar. Pollut. Bull..

[82] Brakstad O.G., Daling P.S., Faksness L.G., Almås I.K., Vang S.H., Syslak L., Leirvik F. (2014). Depletion and biodegradation of hydrocarbons in dispersions and emulsions of the Macondo 252 oil generated in an oil-on-seawater mesocosm flume basin. Mar. Pollut. Bull..

[83] North E.W., Adams E.E., Thessen A.E., Schlag Z., He R., Socolofsky S.A., Masutani S.M., Peckham S.D. (2015). The influence of droplet size and biodegradation on the transport of subsurface oil droplets during the Deepwater Horizon spill: A model sensitivity study. Environ. Res. Lett..

[84] Prince R.C., Nash G.W., Hill S.J. (2016). The biodegradation of crude oil in the deep ocean. Mar. Pollut. Bull..

[85] Head I.M., Jones D.M., Röling W.F.M. (2006). Marine microorganisms make a meal of oil. Nat. Rev. Microbiol..

[86] Bejarano A.C., Levine E., Mearns A.J. (2013). Effectiveness and potential ecological effects of offshore surface dispersant use during the Deepwater Horizon oil spill: A retrospective analysis of monitoring data. Environ. Monit. Assess..

[87] Lee K., Nedwed T., Prince R.C., Palandro D. (2013). Lab tests on the biodegradation of chemically dispersed oil should consider the rapid dilution that occurs at sea. Mar. Pollut. Bull..

[88] Nedwed T., Coolbaugh T. (2008). Do basins and beakers negatively bias dispersant-effectiveness tests?. Int. Oil Spill Conf. Proc..

[89] Banat I.M., Satpute S.K., Cameotra S.S., Patil R., Nyayanit N.V. (2014). Cost effective technologies and renewable substrates for biosurfactants’ production. Front. Microbiol..

[90] Varjani S.J., Upasani V.N. (2017). Critical review on biosurfactant analysis, purification and characterization using rhamnolipid as a model biosurfactant. Bioresour. Technol..

[91] Norman R.S., Frontera-Suau R., Morris P.J. (2002). Variability in *Pseudomonas* aeruginosa lipopolysaccharide expression during crude oil degradation. Appl. Environ. Microbiol..

[92] Kang S.W., Kim Y.B., Shin J.D., Kim E.K. (2010). Enhanced biodegradation of hydrocarbons in soil by microbial biosurfactant, sophorolipid. Appl. Biochem. Biotechnol..

[93] Ashby R.D., Solaiman D.K.Y., Foglia T.A. (2008). Property control of sophorolipids: Influence of fatty acid substrate and blending. Biotechnol. Lett..

[94] Daverey A., Pakshirajan K. (2010). Sophorolipids from Candida bombicola using mixed hydrophilic substrates: Production, purification and characterization. Colloids Surf. B Biointerfaces.

[95] Gregson B.H., Metodieva G., Metodiev M.V., Golyshin P.N., McKew B.A. (2020). Protein expression in the obligate hydrocarbon-degrading psychrophile *Oleispira* antarctica RB-8 during alkane degradation and cold tolerance. Environ. Microbiol..

[96] King G.M., Kostka J.E., Hazen T.C., Sobecky P.A. (2015). Microbial Responses to the Deepwater Horizon Oil Spill: From Coastal Wetlands to the Deep Sea. Ann. Rev. Mar. Sci..

[97] Brakstad O.G., Størseth T.R., Brunsvik A., Bonaunet K., Faksness L.G. (2018). Biodegradation of oil spill dispersant surfactants in cold seawater. Chemosphere.

[98] Krolicka A., Boccadoro C., Mæland M., Preston C., Birch J., Scholin C., Baussant T. Detection of oil leaks by quantifying hydrocarbonoclastic bacteria in cold marine environments using the Environmental Sample Processor. Proceedings of the 37th AMOP Technical Seminar on Environmental Contamination and Response.

[99] Röling W.F.M., Milner M.G., Jones D.M., Lee K., Daniel F., Swannell R.J.P., Head I.M. (2002). Robust hydrocarbon degradation and dynamics of bacterial communities during nutrient-enhanced oil spill bioremediation. Appl. Environ. Microbiol..

[100] Garneau M.É., Michel C., Meisterhans G., Fortin N., King T.L., Greer C.W., Lee K. (2016). Hydrocarbon biodegradation by Arctic sea-ice and sub-ice microbial communities during microcosm experiments, Northwest Passage (Nunavut, Canada). FEMS Microbiol. Ecol..

[101] Yakimov M.M., Giuliano L., Denaro R., Crisafi E., Chernikova T.N., Abraham W.R., Luensdorf H., Timmis K.N., Golyshin P.N. (2004). *Thalassolituus* oleivorans gen. nov., sp. nov., a novel marine bacterium that obligately utilizes hydrocarbons. Int. J. Syst. Evol. Microbiol..

[102] Yakimov M.M., Golyshin P.N., Lang S., Moore E.R.B., Abraham W.R., Lünsdorf H., Timmis K.N. (1998). *Alcanivorax* borkumensis gen. nov., sp. nov., a new, hydrocarbon- degrading and surfactant-producing marine bacterium. Int. J. Syst. Bacteriol..

[103] Schneiker S., Dos Santos V.A.P.M., Bartels D., Bekel T., Brecht M., Buhrmester J., Chernikova T.N., Denaro R., Ferrer M., Gertler C. (2006). Genome sequence of the ubiquitous hydrocarbon-degrading marine bacterium Alcanivorax borkumensis. Nat. Biotechnol..

[104] Gregson B.H., Metodieva G., Metodiev M.V., McKew B.A. (2019). Differential protein expression during growth on linear versus branched alkanes in the obligate marine hydrocarbon-degrading bacterium *Alcanivorax* borkumensis SK2T. Environ. Microbiol..

[105] Cappello S., Yakimov M., McGenity T.J. (2010). Alcanivorax. Handbook of Hydrocarbon and Lipid Microbiology.

[106] Dyksterhouse S.E., Gray J.P., Herwig R.P., Lara J.C., Staley J.T. (1995). *Cycloclasticus* pugetii gen. nov., sp. nov., an Aromatic hydrocarbon- degrading bacterium from marine sediments. Int. J. Syst. Bacteriol..

[107] Wang Y., Lau P.C.K., Button D.K. (1996). A marine oligobacterium harboring genes known to be part of aromatic hydrocarbon degradation pathways of soil pseudomonads. Appl. Environ. Microbiol..

[108] Röling W.F.M., Van Bodegom P.M. (2014). Toward quantitative understanding on microbial community structure and functioning: A modeling-centered approach using degradation of marine oil spills as example. Front. Microbiol..

[109] McGenity T.J., Folwell B.D., McKew B.A., Sanni G.O. (2012). Marine crude-oil biodegradation: A central role for interspecies interactions. Aquat. Biosyst..

[110] Cappello S., Caruso G., Zampino D., Monticelli L.S., Maimone G., Denaro R., Tripodo B., Troussellier M., Yakimov M., Giuliano L. (2007). Microbial community dynamics during assays of harbour oil spill bioremediation: A microscale simulation study. J. Appl. Microbiol..

[111] McKew B.A., Coulon F., Yakimov M.M., Denaro R., Genovese M., Smith C.J., Osborn A.M., Timmis K.N., McGenity T.J. (2007). Efficacy of intervention strategies for bioremediation of crude oil in marine systems and effects on indigenous hydrocarbonoclastic bacteria. Environ. Microbiol..

[112] Noh J., Kim H., Lee C., Yoon S.J., Chu S., Kwon B.O., Ryu J., Kim J.J., Lee H., Yim U.H. (2018). Bioaccumulation of Polycyclic Aromatic Hydrocarbons (PAHs) by the Marine Clam, Mactra veneriformis, Chronically Exposed to Oil-Suspended Particulate Matter Aggregates. Environ. Sci. Technol..

[113] Dombrowski N., Donaho J.A., Gutierrez T., Seitz K.W., Teske A.P., Baker B.J. (2016). Reconstructing metabolic pathways of hydrocarbon-degrading bacteria from the Deepwater Horizon oil spill. Nat. Microbiol..

[114] Suja L.D., Summers S., Gutierrez T. (2017). Role of EPS, dispersant and nutrients on the microbial response and MOS formation in the subarctic Northeast Atlantic. Front. Microbiol..

[115] Naysim L.O., Kang H.J., Jeon C.O. (2014). *Zhongshania* aliphaticivorans sp. nov., an aliphatic hydrocarbon-degrading bacterium isolated from marine sediment, And transfer of Spongiibacter borealis Jang et al. 2011 to the genus *Zhongshania* as *Zhongshania* borealis comb. nov. Int. J. Syst. Evol. Microbiol..

[116] Ribicic D., Netzer R., Hazen T.C., Techtmann S.M., Drabløs F., Brakstad O.G. (2018). Microbial community and metagenome dynamics during biodegradation of dispersed oil reveals potential key-players in cold Norwegian seawater. Mar. Pollut. Bull..

[117] Hubert C.R.J., Oldenburg T.B.P., Fustic M., Gray N.D., Larter S.R., Penn K., Rowan A.K., Seshadri R., Sherry A., Swainsbury R. (2012). Massive dominance of Epsilonproteobacteria in formation waters from a Canadian oil sands reservoir containing severely biodegraded oil. Environ. Microbiol..

[118] Chronopoulou P.M., Sanni G.O., Silas-Olu D.I., van der Meer J.R., Timmis K.N., Brussaard C.P.D., McGenity T.J. (2015). Generalist hydrocarbon-degrading bacterial communities in the oil-polluted water column of the North Sea. Microb. Biotechnol..

[119] Barnsley E.A. (1976). Naphthalene metabolism by pseudomonads: The oxidation of 1,2-dihydroxynaphthalene to 2-hydroxychromene-2-carboxylic acid and the formation of 2′-hydroxybenzalpyruvate. Biochem. Biophys. Res. Commun..

[120] Varjani S.J., Upasani V.N. (2016). Biodegradation of petroleum hydrocarbons by oleophilic strain of *Pseudomonas* aeruginosa NCIM 5514. Bioresour. Technol..

[121] Ramya C., Lakshmi R., Asha D., Sivamurugan V., Vasudevan V., Krishnan M. (2018). Demonstration of bioprocess factors optimization for enhanced mono-rhamnolipid production by a marine *Pseudomonas* guguanensis. Int. J. Biol. Macromol..

[122] Geys R., Soetaert W., Van Bogaert I. (2014). Biotechnological opportunities in biosurfactant production. Curr. Opin. Biotechnol..

[123] Zhang G., Wu Y., Qian X., Meng Q. (2005). Biodegradation of crude oil by *Pseudomonas* aeruginosa in the presence of rhamnolipids. J. Zhejiang Univ. Sci..

[124] Caiazza N.C., Shanks R.M.Q., O’Toole G.A. (2005). Rhamnolipids modulate swarming motility patterns of *Pseudomonas* aeruginosa. J. Bacteriol..

[125] Chrzanowski Ł., Ławniczak Ł., Czaczyk K. (2012). Why do microorganisms produce rhamnolipids?. World J. Microbiol. Biotechnol..

[126] Ibrar M., Zhang H. (2020). Construction of a hydrocarbon-degrading consortium and characterization of two new lipopeptides biosurfactants. Sci. Total Environ..

[127] Marx R.B., Aitken M.D. (2000). Bacterial chemotaxis enhances naphthalene degradation in a heterogeneous aqueous system. Environ. Sci. Technol..

[128] Krell T., Lacal J., Reyes-Darias J.A., Jimenez-Sanchez C., Sungthong R., Ortega-Calvo J.J. (2013). Bioavailability of pollutants and chemotaxis. Curr. Opin. Biotechnol..

[129] Haba E., Pinazo A., Jauregui O., Espuny M.J., Infante M.R., Manresa A. (2003). Physicochemical characterization and antimicrobial properties of rhamnolipids produced by *Pseudomonas* aeruginosa 47T2 NCBIM 40044. Biotechnol. Bioeng..

[130] Kearns D.B. (2010). A field guide to bacterial swarming motility. Nat. Rev. Microbiol..

