# Supplementary material for: Effects of Dispersants and Biosurfactants on Crude-Oil Biodegradation and Bacterial Community Succession
Source: Microorganisms. 2021 Jun 1;9(6):1200. doi: 10.3390/microorganisms9061200 (PMC8229435; doi:10.3390/microorganisms9061200)
Supplement: Supplementary file 1 [file microorganisms-09-01200-s001.zip › Table S1.pdf]

Table S1: Experimental design showing 10 treatments **(codes in parentheses)**; including concentrations and measurements of seawater, nutrients, crude oil (NSO-1), dispersant or surfactants, and, for the killed controls, mercuric chloride (HgCl2).

**Treatment**	**Seawater**	**Nutrients**	**Oil (NSO-1)**	**Surfactant**	**$HgCl_2$**
Seawater & Nutrients<br><br>**(No-Oil Control)**	20 ml	300 µM NH_4Cl,<br>20 µM K_2PO_4			
Seawater, Nutrients, & Oil<br><br>**(Oil-Only)**	20 ml	300 µM NH_4Cl,<br>20 µM K_2PO_4	0.1% v/v		
Slickgone NS<br><br>**(Oil & SG)**	20 ml	300 µM NH_4Cl,<br>20 µM K_2PO_4	0.1% v/v	0.005% v/v	
Superdispersant 25<br><br>**(Oil & SD25)**	20 ml	300 µM NH_4Cl,<br>20 µM K_2PO_4	0.1% v/v	0.005% v/v	
Finasol OSR 52<br><br>**(Oil & F52)**	20 ml	300 µM NH_4Cl,<br>20 µM K_2PO_4	0.1% v/v	0.005% v/v	
Rhamnolipid<br><br>**(Oil & RL)**	20 ml	300 µM NH_4Cl,<br>20 µM K_2PO_4	0.1% v/v	0.005% v/v	
Sophorolipid<br><br>**(Oil & SP)**	20 ml	300 µM NH_4Cl,<br>20 µM K_2PO_4	0.1% v/v	0.005% v/v	
Trehalolipid<br><br>**(Oil & TH)**	20 ml	300 µM NH_4Cl,<br>20 µM K_2PO_4	0.1% v/v	0.005% v/v	
Killed Control<br><br>**(Kill)**	20 ml		0.1% v/v		1.1049 mM
