# Supplementary material for: Effects of Dispersants and Biosurfactants on Crude-Oil Biodegradation and Bacterial Community Succession
Source: Microorganisms. 2021 Jun 1;9(6):1200. doi: 10.3390/microorganisms9061200 (PMC8229435; doi:10.3390/microorganisms9061200)
Supplement: Supplementary file 1 [file microorganisms-09-01200-s001.zip › Table S2.pdf]

Table. S2: Evidence demonstrating hydrocarbon degradation (alkane of PAH) and growth in oil-polluted marine environments for certain genera of Bacteria (either so-called "obligate hydrocarbonoclastic Bacteria" or more metabolically versatile).

Genus	Hydrocarbon Degradation Metabolic Capability	Isolated Alkane Degraders	Isolated Polycyclic Aromatic Hydrocarbon Degraders	Evidence for Increased Abundance in Oiled Marine Environments*
Alcanivorax spp.	OHCB*	**Yes**<br>Yakimov *et al.*, 1998	**No**<br>May enhance PAH degradation utilising alkyl sidechains on methylated PAHs<br>(Yuan *et al.*, 2015; Noh *et al.*, 2018)	**Yes**<br>Kasai *et al.*, 2001;<br>Röling *et al.*, 2004;<br>McKew *et al.*, 2007;<br>Wang *et al.*, 2010;<br>Teramoto *et al.*, 2013;<br>Sanni *et al.*, 2015;<br>Lee *et al.*, 2017;<br>Liu *et al.*, 2019
Alkalimarinus spp.	Versatile±	**No**	**No**	**Yes**<br>Noh *et al.*, 2018;<br>Li *et al.*, 2019
Arcobacter spp.	Versatile±	**No**	**No**<br>Potential for benzene degradation has been observed within *in-situ* microcosms in constructed wetlands (Nitz *et al.*, 2019)	**Yes**<br>Coulon *et al.*, 2007;<br>Hubert *et al.*, 2012;<br>Isaac *et al.*, 2013;<br>Wang *et al.*, 2014;<br>Lormières and Oger, 2017
Colwellia spp.	Versatile±	**No**	**Yes**<br>"Crude Oil" Bælum et al., 2012<br>Gutierrez *et al.*, 2013<br>Mason *et al.*, 2014	**Yes**<br>Brakstad *et al.*, 2008;<br>Redmond *et al.*, 2010;<br>Dubinsky *et al.*, 2013;<br>Bacosa *et al.*, 2018;<br>Tremblay *et al.*, 2019
Cycloclasticus spp.	OHCB*	**No**<br>Provision – *Cycloclasticus* sp. symbiont of *Bathymodiolus heckerae* (Rubin-Blum *et al.*, 2017)	**Yes**<br>Dyksterhouse *et al.*, 1995<br>Niepceron *et al.*, 2010	**Yes**<br>Coulon *et al.*, 2007;<br>Dubinsky *et al.*, 2013;<br>Sanni *et al.*, 2015;<br>Lee *et al.*, 2017;<br>Liu *et al.*, 2017;<br>Linda *et al.*, 2018;<br>Tremblay *et al.*, 2019
Glaciecola spp.	Versatile±	**Yes**<br>Chronopoulou *et al.*, 2015	**Yes**<br>Chronopoulou *et al.*, 2015	**Yes**<br>Brakstad *et al.*, 2008;<br>Gontikaki *et al.*, 2018;<br>Tremblay *et al.*, 2019

Marinomonas spp.	Versatile±	**No**	**Yes**<br>Melcher *et al.*, 2002	**Yes**<br>Brakstad *et al.*, 2008;<br>Alonso-Gutiérrez *et al.*, 2008;<br>Dong *et al.*, 2015;<br>Gontikaki *et al.*, 2018
Neptuniibacter spp.	Versatile±	**No**	**Yes**<br>Nagashima *et al.*, 2010	**Yes**<br>Rivers *et al.*, 2013;<br>Dombrowski *et al.*, 2016;<br>Doyle *et al.*, 2018;<br>Krolicka *et al.*, 2019
Oleibacter spp.	OHCB*	**Yes**<br>Teramoto *et al.*, 2011	**No**	**Yes**<br>Teramoto *et al.*, 2013;<br>Sanni *et al.*, 2015;<br>Liu *et al.*, 2017;<br>Liu *et al.*, 2019
Oleispira spp.	OHCB*	**Yes**<br>Yakimov *et al.*, 2003	**No**	**Yes**<br>Coulon *et al.*, 2007;<br>King *et al.*, 2015;<br>Boccadoro *et al.*, 2018;<br>Brakstad *et al.*, 2018;<br>Tremblay *et al.*, 2019
Pseudoalteromonas spp.	Versatile±	**Yes**<br>Chronopoulou *et al.*, 2015	**Yes**<br>Chronopoulou *et al.*, 2015	**Yes**<br>Kostka *et al.*, 2011;<br>Dong *et al.*, 2015;<br>Gontikaki *et al.*, 2018;<br>Tremblay et al., 2019
Pseudomonas spp.	Versatile±	**Yes**<br>van Beilen *et al.*, 2001<br>Zhang *et al.*, 2011	**Yes**<br>Niepceron *et al.*, 2010<br>Zhang *et al.*, 2011<br>Chebbi *et al.*, 2017	**Yes**<br>Das and Mukherjee, 2007;<br>Kostka *et al.*, 2011;<br>Dubinsky *et al.*, 2013;<br>Dong *et al.*, 2015;<br>Tremblay *et al.*, 2019
Thalassolituus spp.	OHCB*	**Yes**<br>Yakimov *et al.*, 2004	**No**	**Yes**<br>McKew *et al.*, 2007;<br>Sanni *et al.*, 2015;<br>Lee *et al.*, 2017;<br>Liu *et al.*, 2019
Zhongshania spp.	Versatile±	**Yes**<br>Naysim *et al.*, 2014	**No**	**Yes**<br>Ribicic *et al.*, 2018

* **OHCB** – refers to the so-called “Obligate Hydrocarbonoclastic Bacteria” (Yakimov *et al.*, 2007)
± **Versatile** – has the metabolic capabilities to grow on a diverse range of substrates
* These lists are by no means exhaustive but are examples of the growth of these genera in either *in situ* or *ex situ* oil-based systems.

References

Alonso-Gutiérrez, J., Costa, M.M., Figueras, A., Albaigés, J., Viñas, M., Solanas, A.M., and Novoa, B. (2008) Alcanivorax strain detected among the cultured bacterial community from sediments affected by the “Prestige” oil spill. *Mar Ecol Prog Ser* **362**: 25–36.

Bacosa, H.P., Erdner, D.L., Rosenheim, B.E., Shetty, P., Seitz, K.W., Baker, B.J., and Liu, Z. (2018) Hydrocarbon degradation and response of seafloor sediment bacterial community in the northern Gulf of Mexico to light Louisiana sweet crude oil. *ISME J* **12**: 2532–2543.

Bælum, J., Borglin, S., Chakraborty, R., Fortney, J.L., Lamendella, R., Mason, O.U., et al. (2012) Deep-sea bacteria enriched by oil and dispersant from the Deepwater Horizon spill. *Environ Microbiol* **14**: 2405–2416.

van Beilen, J.B., Panke, S., Lucchini, S., Franchini, A.G., Röthlisberger, M., and Witholt, B. (2001) Analysis of Pseudomonas putida alkane-degradation gene clusters and flanking insertion sequences: Evolution and regulation of the alk genes. *Microbiology* **147**: 1621–1630.

Boccadoro, C., Krolicka, A., Receveur, J., Aeppli, C., and Le Floch, S. (2018) Microbial community response and migration of petroleum compounds during a sea-ice oil spill experiment in Svalbard. *Mar Environ Res* **142**: 214–233.

Brakstad, O.G., Nonstad, I., Faksness, L.G., and Brandvik, P.J. (2008) Responses of microbial communities in Arctic sea ice after contamination by crude petroleum oil. *Microb Ecol* **55**: 540–552.

Brakstad, O.G., Ribicic, D., Winkler, A., and Netzer, R. (2018) Biodegradation of dispersed oil in seawater is not inhibited by a commercial oil spill dispersant. *Mar Pollut Bull* **129**: 555–561.

Chebbi, A., Hentati, D., Zaghden, H., Baccar, N., Rezgui, F., Chalbi, M., et al. (2017) Polycyclic aromatic hydrocarbon degradation and biosurfactant production by a newly isolated

Pseudomonas sp. strain from used motor oil-contaminated soil. *Int Biodeterior Biodegrad* **122**: 128–140.

Chronopoulou, P.M., Sanni, G.O., Silas-Olu, D.I., van der Meer, J.R., Timmis, K.N., Brussaard, C.P.D., and McGenity, T.J. (2015) Generalist hydrocarbon-degrading bacterial communities in the oil-polluted water column of the North Sea. *Microb Biotechnol* **8**: 434–447.

Coulon, F., McKew, B.A., Osborn, A.M., McGenity, T.J., and Timmis, K.N. (2007) Effects of temperature and biostimulation on oil-degrading microbial communities in temperate estuarine waters. *Environ Microbiol* **9**: 177–186.

Das, K. and Mukherjee, A.K. (2007) Crude petroleum-oil biodegradation efficiency of Bacillus subtilis and Pseudomonas aeruginosa strains isolated from a petroleum-oil contaminated soil from North-East India. *Bioresour Technol* **98**: 1339–1345.

Dombrowski, N., Donaho, J.A., Gutierrez, T., Seitz, K.W., Teske, A.P., and Baker, B.J. (2016) Reconstructing metabolic pathways of hydrocarbon-degrading bacteria from the Deepwater Horizon oil spill. *Nat Microbiol* **1**: 1–7.

Dong, C., Bai, X., Sheng, H., Jiao, L., Zhou, H., and Shao, Z. (2015) Distribution of PAHs and the PAH-degrading bacteria in the deep-sea sediments of the high-latitude Arctic Ocean. *Biogeosciences* **12**: 2163–2177.

Doyle, S.M., Whitaker, E.A., De Pascuale, V., Wade, T.L., Knap, A.H., Santschi, P.H., et al. (2018) Rapid formation of microbe-oil aggregates and changes in community composition in coastal surface water following exposure to oil and the dispersant corexit. *Front Microbiol* **9**: 1–16.

Dubinsky, E.A., Conrad, M.E., Chakraborty, R., Bill, M., Borglin, S.E., Hollibaugh, J.T., et al. (2013) Succession of hydrocarbon-degrading bacteria in the aftermath of the deepwater horizon oil spill in the gulf of Mexico. *Environ Sci Technol* **47**: 10860–10867.

Dyksterhouse, S.E., GRAY, J.P., HERWIG, R.P., LARA, J.C., and STALEY, J.T. (1995) Cycloclasticus pugetti gen. nov., sp. nov., an aromatic hydrocarbon-degrading bacterium from marine sediments. *Int J Syst Bacteriol* **45**: 116-123.

Gontikaki, E., Potts, L.D., Anderson, J.A., and Witte, U. (2018) Hydrocarbon-degrading bacteria in deep-water subarctic sediments (Faroe-Shetland Channel). *J Appl Microbiol* **125**: 1040–1053.

Gutierrez, T., Singleton, D.R., Berry, D., Yang, T., Aitken, M.D., and Teske, A. (2013) Hydrocarbon-degrading bacteria enriched by the Deepwater Horizon oil spill identified by cultivation and DNA-SIP. *ISME J* **7**: 2091–2104.

Hubert, C.R.J., Oldenburg, T.B.P., Fustic, M., Gray, N.D., Larter, S.R., Penn, K., et al. (2012) Massive dominance of Epsilonproteobacteria in formation waters from a Canadian oil sands reservoir containing severely biodegraded oil. *Environ Microbiol* **14**: 387–404.

Isaac, P., Sánchez, L.A., Bourguignon, N., Cabral, M.E., and Ferrero, M.A. (2013) Indigenous PAH-degrading bacteria from oil-polluted sediments in Caleta Cordova, Patagonia Argentina. *Int Biodeterior Biodegrad* **82**: 207–214.

Kasai, Y., Kishira, H., Syutsubo, K., and Harayama, S. (2001) Molecular detection of marine bacterial populations on beaches contaminated by the Nakhodka tanker oil-spill accident. *Environ Microbiol* **3**: 246–255.

King, G.M., Kostka, J.E., Hazen, T.C., and Sobecky, P.A. (2015) Microbial Responses to the Deepwater Horizon Oil Spill: From Coastal Wetlands to the Deep Sea. *Ann Rev Mar Sci* **7**: 377–401.

Kostka, J.E., Prakash, O., Overholt, W.A., Green, S.J., Freyer, G., Canion, A., et al. (2011) Hydrocarbon-degrading bacteria and the bacterial community response in Gulf of Mexico beach sands impacted by the deepwater horizon oil spill. *Appl Environ Microbiol* **77**: 7962–7974.

Krolicka, A., Boccadoro, C., Nilsen, M.M., Demir-Hilton, E., Birch, J., Preston, C., et al. (2019) Identification of microbial key-indicators of oil contamination at sea through tracking of oil biotransformation: An Arctic field and laboratory study. *Sci Total Environ* **696**:.

Lee, J., Han, I., Kang, B.R., Kim, S.H., Sul, W.J., and Lee, T.K. (2017) Degradation of crude oil in a contaminated tidal flat area and the resilience of bacterial community. *Mar Pollut Bull* **114**: 296–301.

Linda, A., Hernado, P.B., and Yuewen, D. (2018) Response of microbial communities to oil spill in the Gulf of Mexico: A review. *African J Microbiol Res* **12**: 536–545.

Liu, J., Bacosa, H.P., and Liu, Z. (2017) Potential environmental factors affecting oil-degrading bacterial populations in deep and surface waters of the Northern Gulf of Mexico. *Front Microbiol* **7**: 1–14.

Liu, J., Zheng, Y., Lin, H., Wang, X., Li, M., Liu, Y., et al. (2019) Proliferation of hydrocarbon-degrading microbes at the bottom of the Mariana Trench. *Microbiome* **7**: 1–13.

Lormières, F. and Oger, P.M. (2017) Epsilonproteobacteria dominate bacterial diversity at a natural tar seep. *Comptes Rendus - Biol* **340**: 238–243.

Lozada, M., Marcos, M.S., Commendatore, M.G., Gil, M.N., and Dionisi, H.M. (2014) The Bacterial Community Structure of Hydrocarbon-Polluted Marine Environments as the Basis for the Definition of an Ecological Index of Hydrocarbon Exposure. *Microbes Environ* **29**: 269–276.

Mason, O.U., Han, J., Woyke, T., and Jansson, J.K. (2014) Single-cell genomics reveals features of a Colwellia species that was dominant during the Deepwater Horizon oil spill. *Front Microbiol* **5**:.

Melcher, R.J., Apitz, S.E., and Hemmingsen, B.B. (2002) Impact of irradiation and polycyclic aromatic hydrocarbon spiking on microbial populations in marine sediment for future aging and biodegradability studies. *Appl Environ Microbiol* **68**: 2858–2868.

Nagashima, H., Zulkharnain, A. Bin, Maeda, R., Fuse, H., Iwata, K., and Omori, T. (2010) Cloning and Nucleotide Sequences of Carbazole Degradation Genes from Marine Bacterium Neptuniibacter sp. Strain CAR-SF. *Curr Microbiol* **61**: 50–56.

Naysim, L.O., Kang, H.J., and Jeon, C.O. (2014) Zhongshania aliphaticivorans sp. nov., an aliphatic hydrocarbon-degrading bacterium isolated from marine sediment, And transfer of Spongiibacter borealis Jang et al. 2011 to the genus Zhongshania as Zhongshania borealis comb. nov. *Int J Syst Evol Microbiol* **64**: 3768–3774.

Niepceron, M., Portet-Koltalo, F., Merlin, C., Motelay-Massei, A., Barray, S., and Bodilis, J. (2010) Both Cycloclasticus spp. and Pseudomonas spp. as PAH-degrading bacteria in the Seine estuary (France). *FEMS Microbiol Ecol* **71**: 137–147.

Nitz, H., Duarte, M., Jauregui, R., Pieper, D.H., Müller, J.A., and Kästner, M. (2020) Identification of benzene-degrading Proteobacteria in a constructed wetland by employing in situ microcosms and RNA-stable isotope probing. *Appl Microbiol Biotechnol* **104**: 1809–1820.

Noh, J., Kim, H., Lee, C., Yoon, S.J., Chu, S., Kwon, B.O., et al. (2018) Bioaccumulation of Polycyclic Aromatic Hydrocarbons (PAHs) by the Marine Clam, Mactra veneriformis, Chronically Exposed to Oil-Suspended Particulate Matter Aggregates. *Environ Sci Technol* **52**: 7910–7920.

Redmond, M.C., Valentine, D.L., and Sessions, A.L. (2010) Identification of novel methane-, ethane-, and propane-oxidizing bacteria at marine hydrocarbon seeps by stable isotope probing. *Appl Environ Microbiol* **76**: 6412–6422.

Ribicic, D., Netzer, R., Winkler, A., and Brakstad, O.G. (2018) Microbial communities in seawater from an Arctic and a temperate Norwegian fjord and their potentials for biodegradation of chemically dispersed oil at low seawater temperatures. *Mar Pollut Bull* **129**: 308–317.

Rivers, A.R., Sharma, S., Tringe, S.G., Martin, J., Joye, S.B., and Moran, M.A. (2013)

Transcriptional response of bathypelagic marine bacterioplankton to the Deepwater Horizon oil spill. *ISME J* **7**: 2315–2329.

Röling, W.F.M., Milner, M.G., Jones, D.M., Fratepietro, F., Swannell, R.P.J., Daniel, F., and Head, I.M. (2004) Bacterial community dynamics and hydrocarbon degradation during a field-scale evaluation of bioremediation on a mudflat beach contaminated with buried oil. *Appl Environ Microbiol* **70**: 2603–2613.

Rubin-Blum, M., Antony, C.P., Borowski, C., Sayavedra, L., Pape, T., Sahling, H., et al. (2017) Short-chain alkanes fuel mussel and sponge Cycloclasticus symbionts from deep-sea gas and oil seeps. *Nat Microbiol* **2**:.

Sanni, G.O., Coulon, F., and McGenity, T.J. (2015) Dynamics and distribution of bacterial and archaeal communities in oil-contaminated temperate coastal mudflat mesocosms. *Environ Sci Pollut Res* **22**: 15230–15247.

Teramoto, M., Ohuchi, M., Hatmanti, A., Darmayati, Y., Widyastuti, Y., Harayama, S., and Fukunaga, Y. (2011) Oleibacter marinus gen. nov., sp. nov., a bacterium that degrades petroleum aliphatic hydrocarbons in a tropical marine environment. *Int J Syst Evol Microbiol* **61**: 375–380.

Teramoto, M., Queck, S.Y., and Ohnishi, K. (2013) Specialized Hydrocarbonoclastic Bacteria Prevailing in Seawater around a Port in the Strait of Malacca. *PLoS One* **8**: 2–8.

Tremblay, J., Fortin, N., Elias, M., Wasserscheid, J., King, T.L., Lee, K., and Greer, C.W. (2019) Metagenomic and metatranscriptomic responses of natural oil degrading bacteria in the presence of dispersants. *Environ Microbiol* **21**: 2307–2319.

Wang, L.Y., Ke, W.J., Sun, X.B., Liu, J.F., Gu, J.D., and Mu, B.Z. (2014) Comparison of bacterial community in aqueous and oil phases of water-flooded petroleum reservoirs using pyrosequencing and clone library approaches. *Appl Microbiol Biotechnol* **98**: 4209–4221.

Wang, W., Wang, L., and Shao, Z. (2010) Diversity and Abundance of Oil-Degrading Bacteria

and Alkane Hydroxylase (alkB) Genes in the Subtropical Seawater of Xiamen Island. *Microb Ecol* **60**: 429–439.

Yakimov, M.M., Giuliano, L., Denaro, R., Crisafi, E., Chernikova, T.N., Abraham, W.R., et al. (2004) Thalassolituus oleivorans gen. nov., sp. nov., a novel marine bacterium that obligately utilizes hydrocarbons. *Int J Syst Evol Microbiol* **54**: 141–148.

Yakimov, M.M., Giuliano, L., Gentile, G., Crisafi, E., Chernikova, T.N., Abraham, W.R., et al. (2003) Oleispira antarctica gen. nov., sp. nov., a novel hydrocarbonoclastic marine bacterium isolated from Antarctic coastal sea water. *Int J Syst Evol Microbiol* **53**: 779–785.

Yakimov, M.M., Golyshin, P.N., Lang, S., Moore, E.R.B., Abraham, W.R., Lünsdorf, H., and Timmis, K.N. (1998) Alcanivorax borkumensis gen. nov., sp. nov., a new, hydrocarbon-degrading and surfactant-producing marine bacterium. *Int J Syst Bacteriol* **48**: 339–348.

Yakimov, M.M., Timmis, K.N., and Golyshin, P.N. (2007) Obligate oil-degrading marine bacteria. *Curr Opin Biotechnol* **18**: 257–266.

Yuan, J., Lai, Q., Sun, F., Zheng, T., and Shao, Z. (2015) The diversity of PAH-degrading bacteria in a deep-sea water column above the southwest Indian ridge. *Front Microbiol* **6**: 1–12.

Zhang, Z., Hou, Z., Yang, C., Ma, C., Tao, F., and Xu, P. (2011) Degradation of n-alkanes and polycyclic aromatic hydrocarbons in petroleum by a newly isolated Pseudomonas aeruginosa DQ8. *Bioresour Technol* **102**: 4111–4116.
