# Supplementary figures and images for: Effects of Dispersants and Biosurfactants on Crude-Oil Biodegradation and Bacterial Community Succession
Source: Microorganisms. 2021 Jun 1;9(6):1200. doi: 10.3390/microorganisms9061200 (PMC8229435; doi:10.3390/microorganisms9061200)

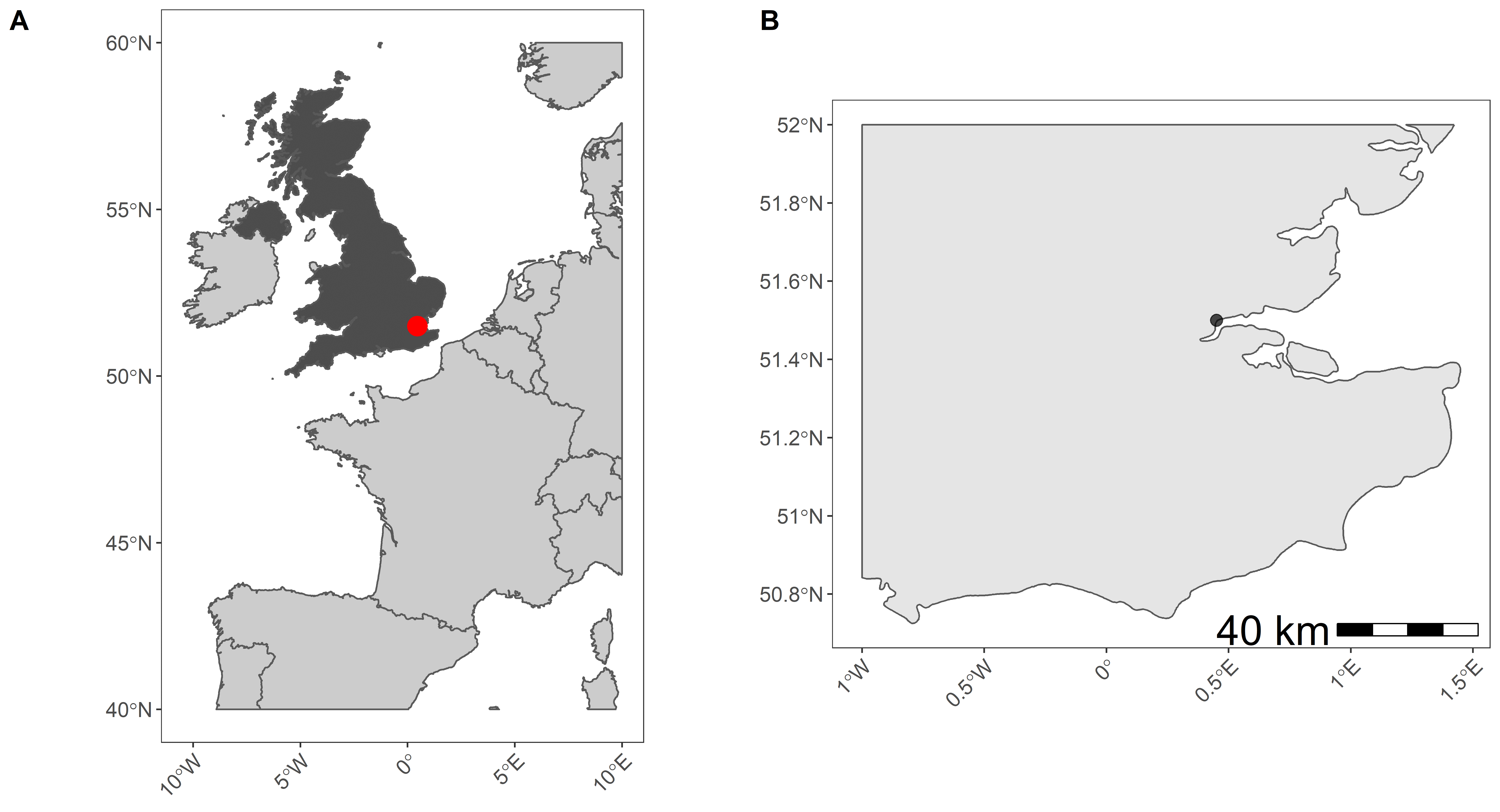

Supplement: Supplementary file 1 [file microorganisms-09-01200-s001.zip › Figure S1.png]

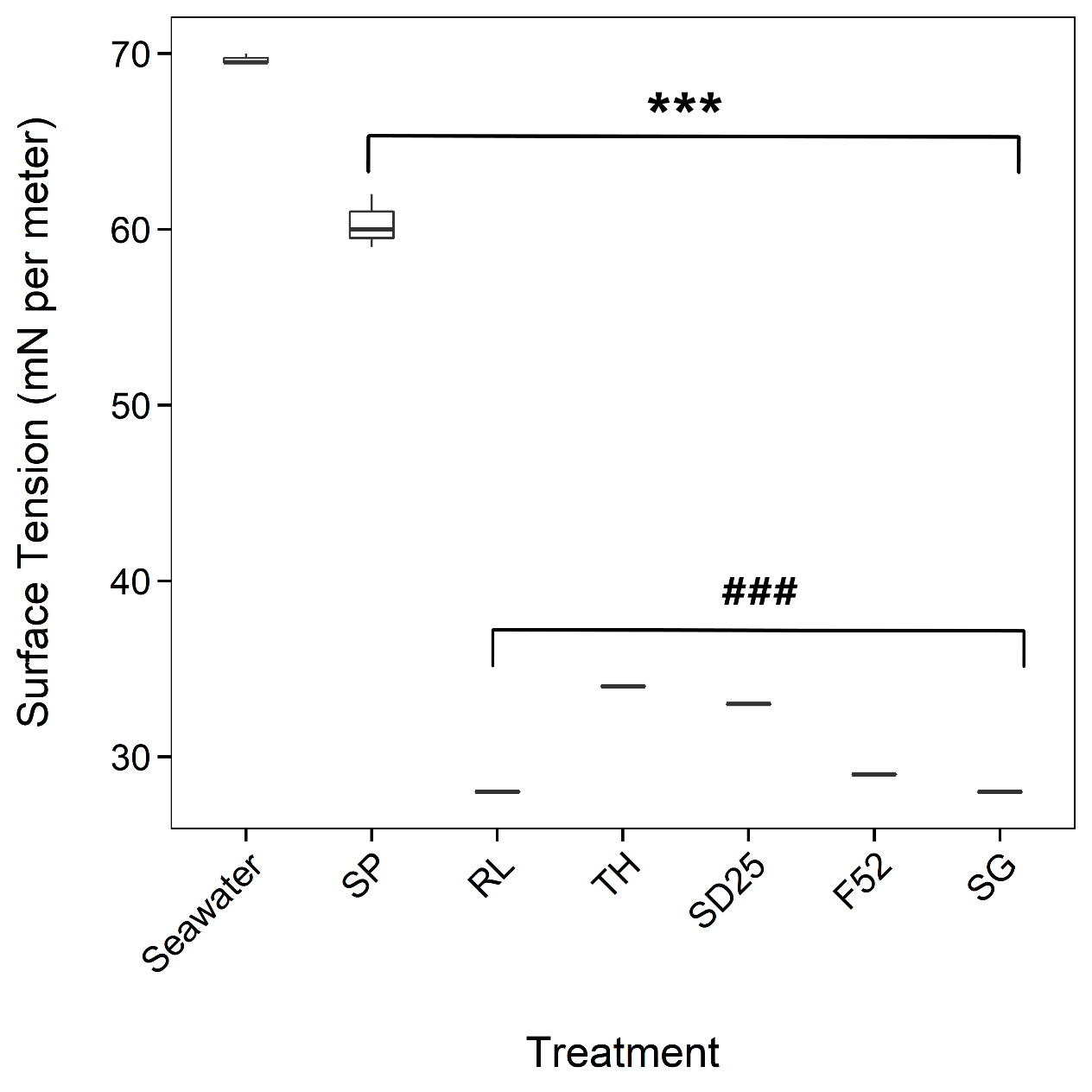

Supplement: Supplementary file 1 [file microorganisms-09-01200-s001.zip › Figure S2.png]

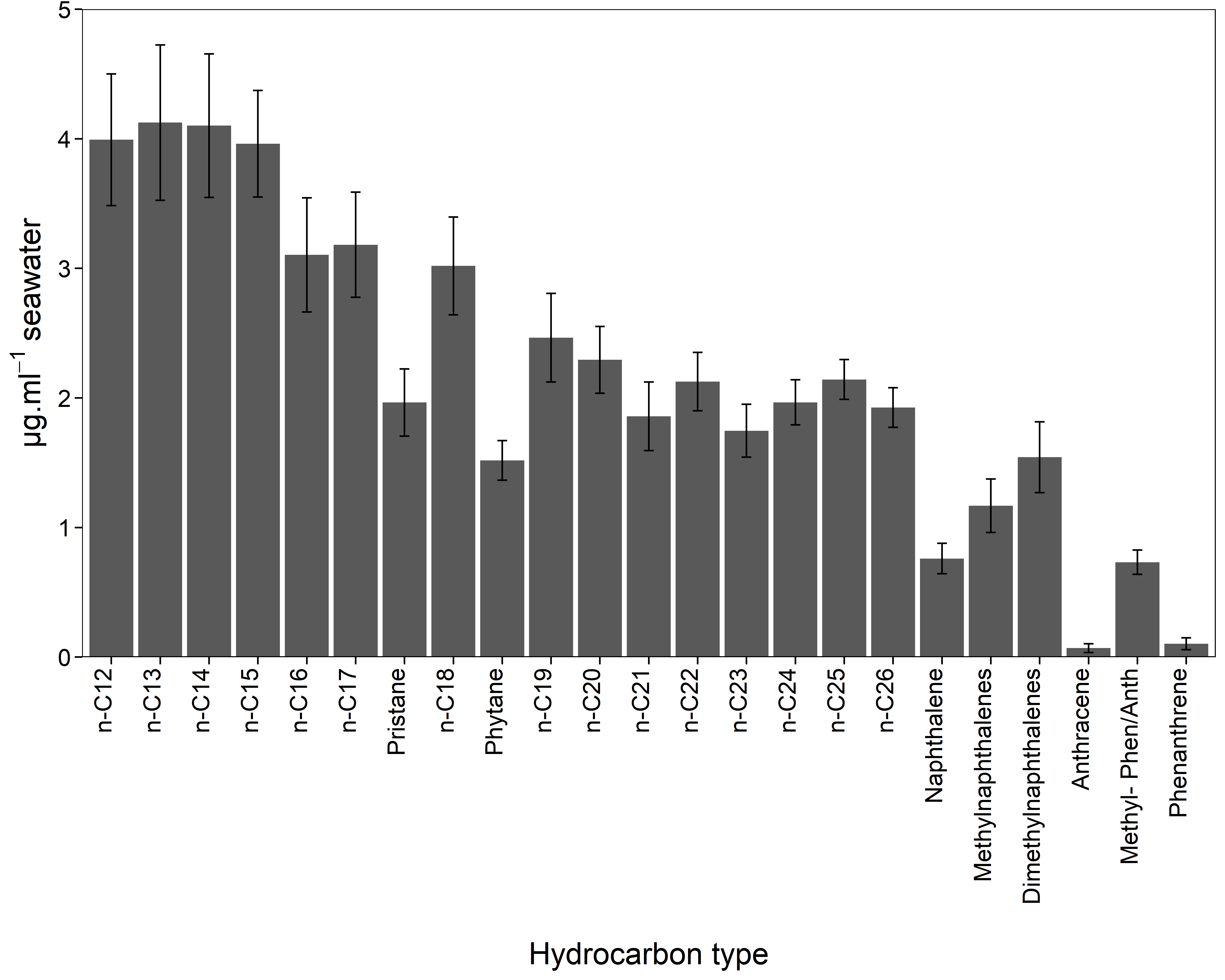

Supplement: Supplementary file 1 [file microorganisms-09-01200-s001.zip › Figure S3.png]

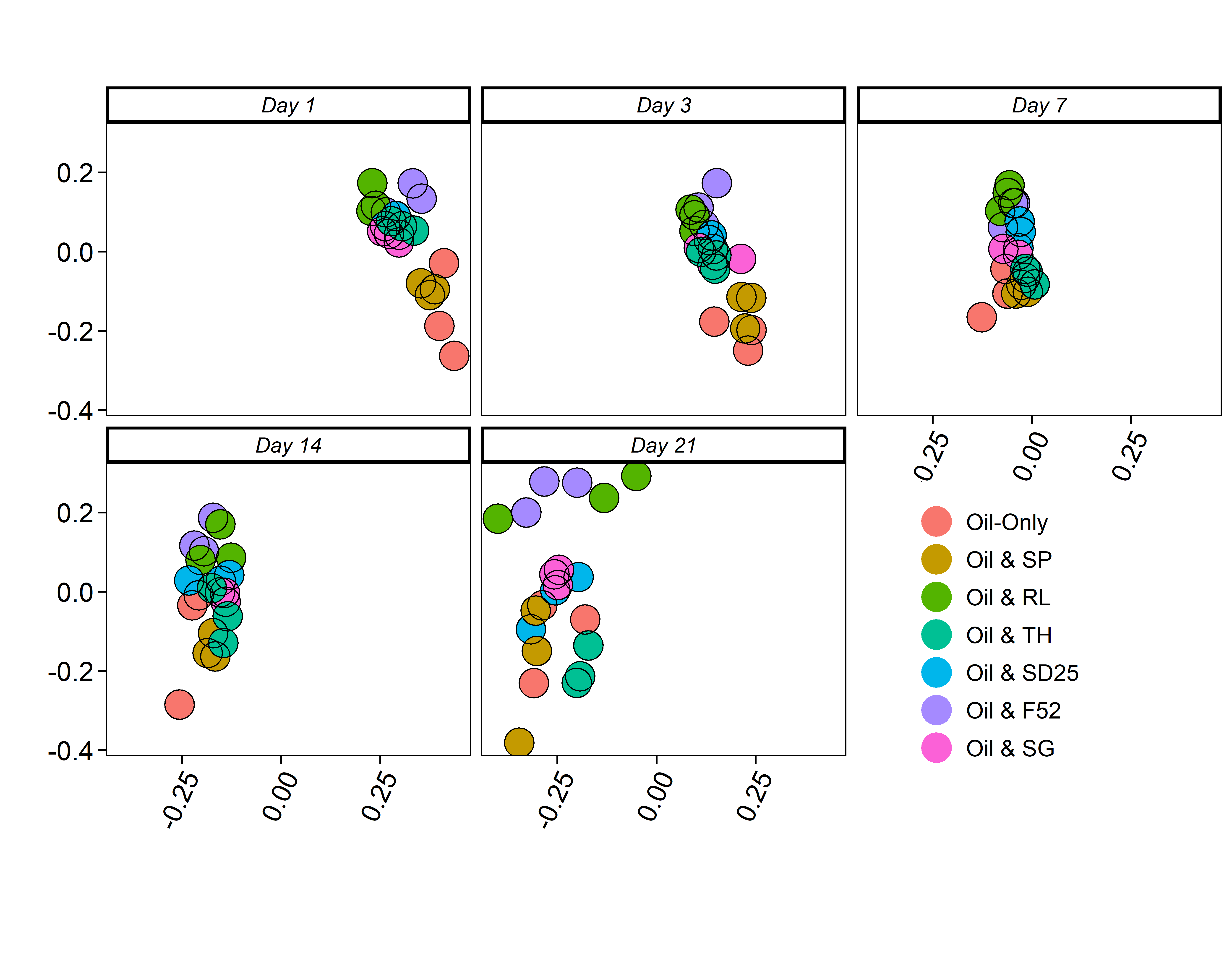

Supplement: Supplementary file 1 [file microorganisms-09-01200-s001.zip › Figure S4.png]

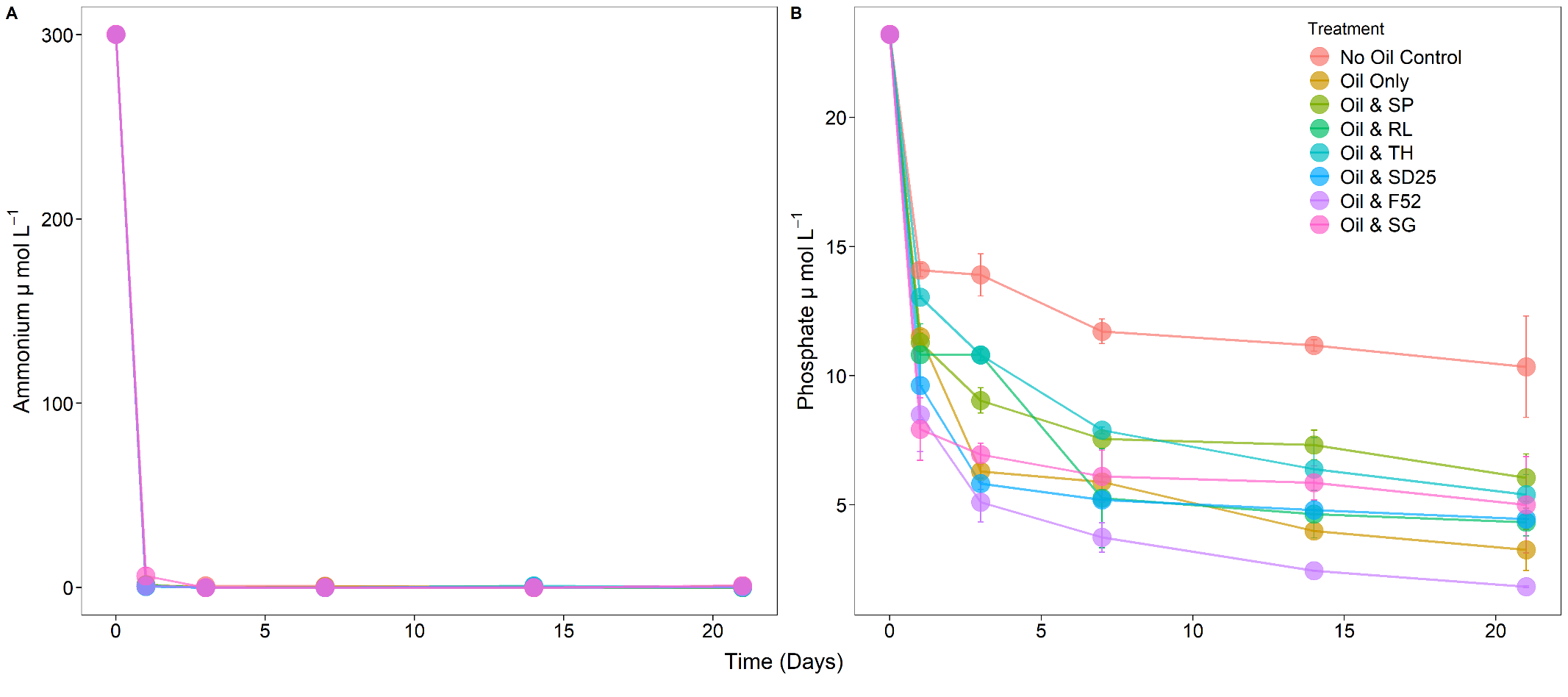

Supplement: Supplementary file 1 [file microorganisms-09-01200-s001.zip › Figure S5.png]

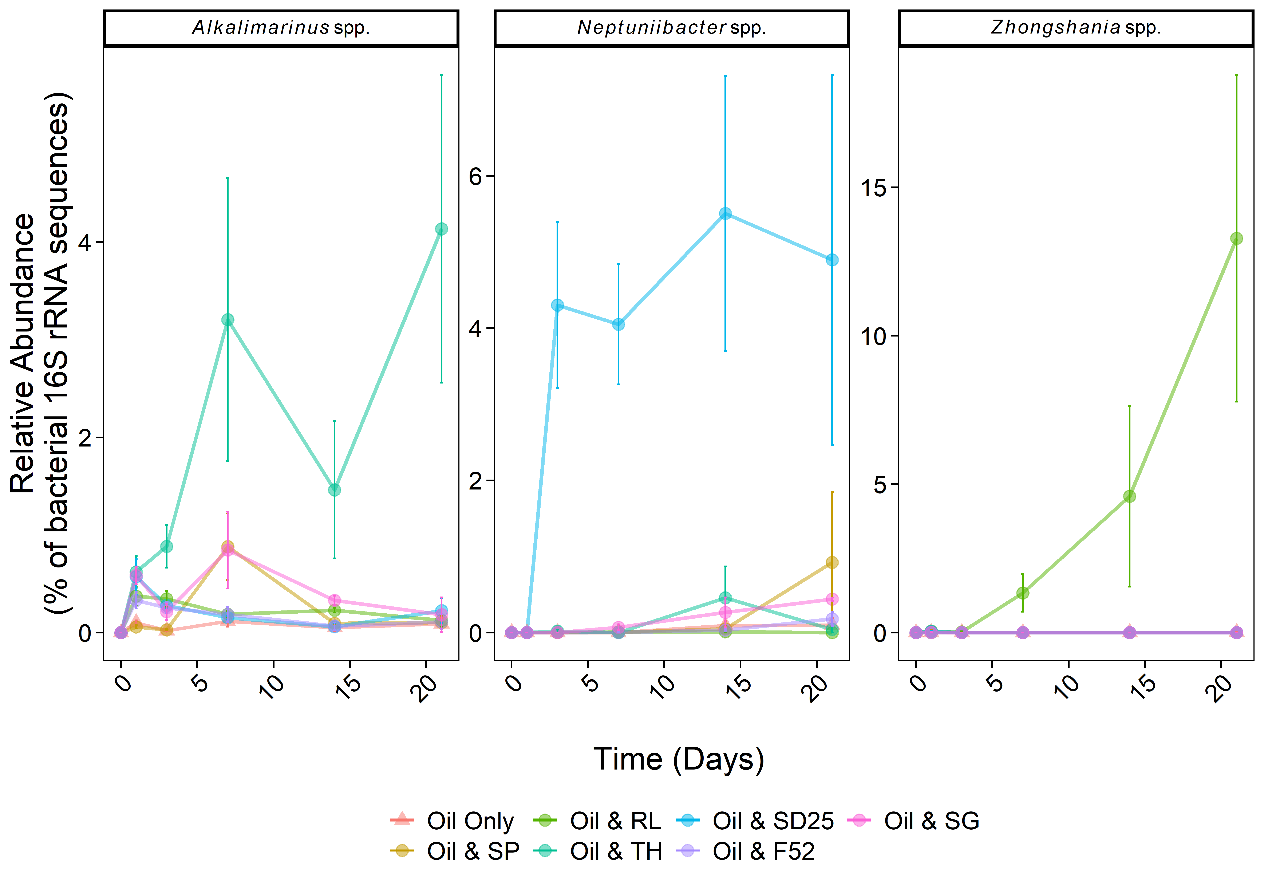

Supplement: Supplementary file 1 [file microorganisms-09-01200-s001.zip › Figure S6.png]

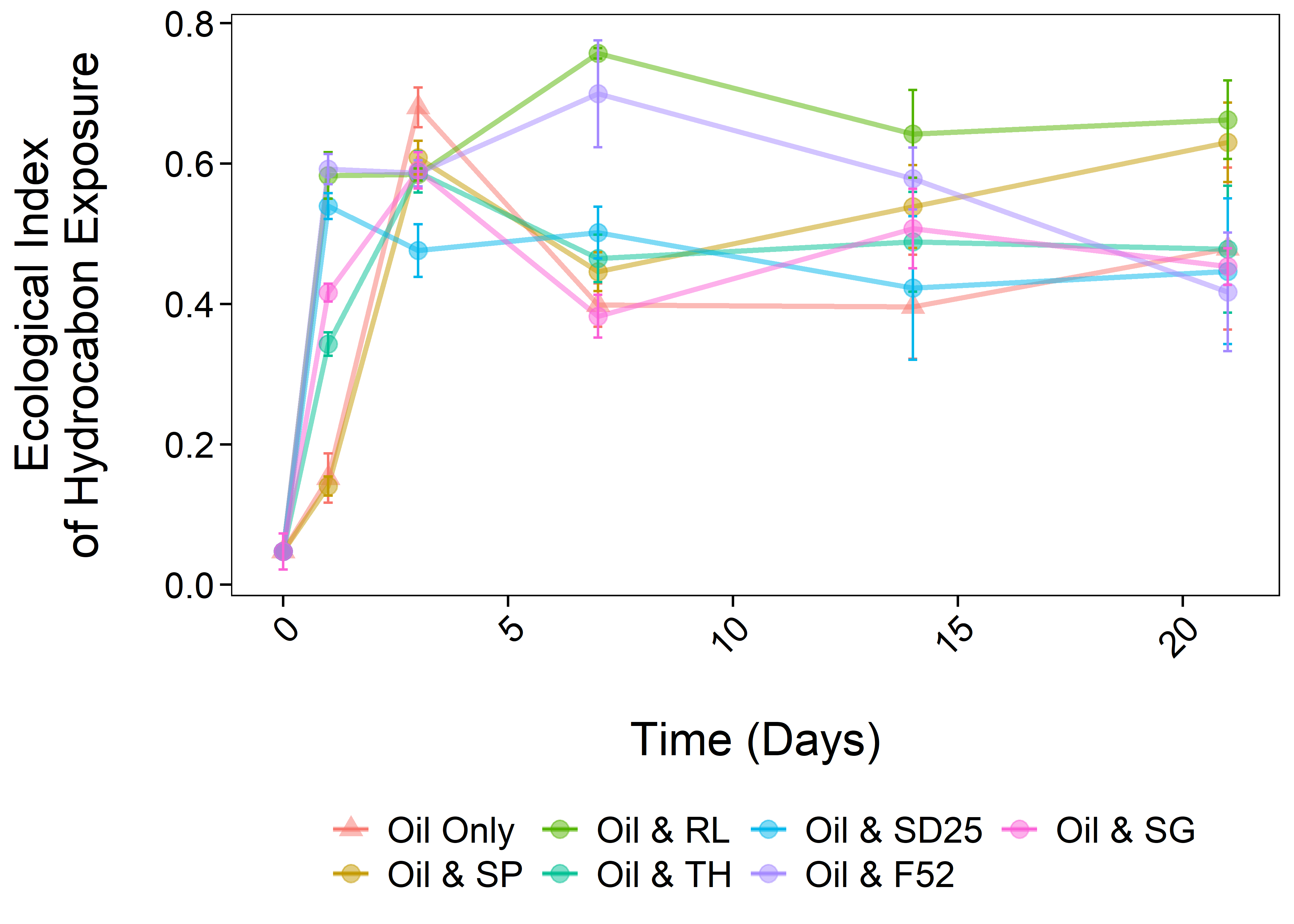

Supplement: Supplementary file 1 [file microorganisms-09-01200-s001.zip › Figure S7.png]

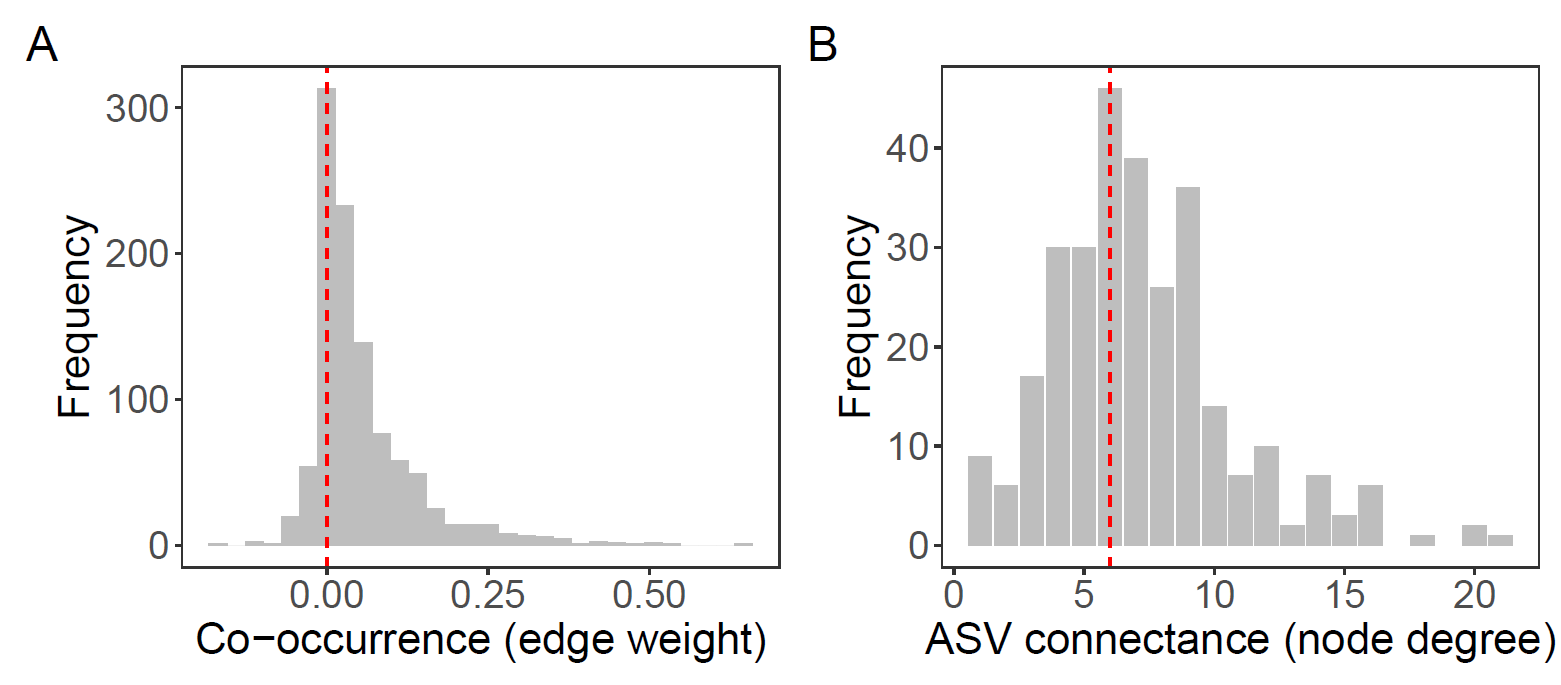

Supplement: Supplementary file 1 [file microorganisms-09-01200-s001.zip › Figure S8.png]

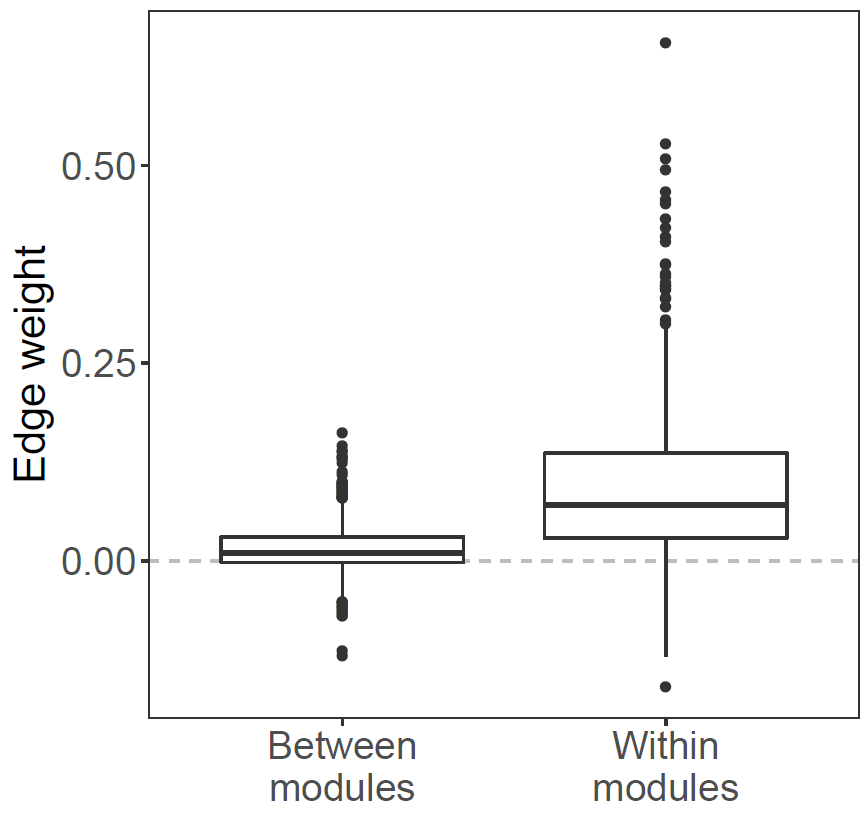

Supplement: Supplementary file 1 [file microorganisms-09-01200-s001.zip › Figure S9.png]
